# Two-Photon Absorption Cooperative Effects within Multi-Dipolar Ruthenium Complexes: The Decisive Influence of Charge Transfers

**DOI:** 10.3390/molecules27051493

**Published:** 2022-02-23

**Authors:** Nicolas Durand, Anissa Amar, Rana Mhanna, Huriye Akdas-Kiliç, Olivier Soppera, Jean-Pierre Malval, Abdou Boucekkine, Jean-Luc Fillaut

**Affiliations:** 1 Univ. Rennes, CNRS, ISCR (Institut des Sciences Chimiques de Rennes) UMR 6226, 35000 Rennes, France; nicolaval@hotmail.com (N.D.); anissa.amar@ummto.dz (A.A.); huriye.akdas@univ-rennes1.fr (H.A.-K.); 2 Laboratoire de Physique et Chimie Quantiques, Faculté des Sciences, Université Mouloud Mammeri de Tizi-Ouzou, Tizi-Ouzou 15000, Algeria; 3 Institut de Science des Matériaux de Mulhouse CNRS-UMR 7361, Université de Haute Alsace, 15 rue Jean Starcky, 68057 Mulhouse, France; rana.mhanna@uha.fr (R.M.); olivier.soppera@uha.fr (O.S.); jean-pierre.malval@uha.fr (J.-P.M.); 4 Department of Chemistry, Yildiz Technical University, Esenler, Istanbul 34220, Turkey

**Keywords:** coordination chemistry, ruthenium polypyridyl complexes, two-photon absorption, DFT computations, charge transfer, cooperative effect

## Abstract

One- and two-photon characterizations of a series of hetero- and homoleptic [RuL_3-n_(bpy)_n_]^2+^ (*n* = 0, 1, 2) complexes carrying bipyridine π-extended ligands (L), have been carried out. These π-extended D−π−A−A−π−D-type ligands (L), where the electron donor units (D) are based on diphenylamine, carbazolyl, or fluorenyl units, have been designed to modulate the conjugation extension and the donating effect. Density functional theory calculations were performed in order to rationalize the observed spectra. Calculations show that the electronic structure of the π-extended ligands has a pronounced effect on the composition of HOMO and LUMO and on the metallic contribution to frontier MOs, resulting in strikingly different nonlinear properties. This work demonstrates that ILCT transitions are the keystone of one- and two-photon absorption bands in the studied systems and reveals how much MLCT and LLCT charge transfers play a decisive role on the two-photon properties of both hetero- and homoleptic ruthenium complexes through cooperative or suppressive effects.

## 1. Introduction

Over the past two decades, increasing attention has been devoted to the design and study of molecular materials with optimized two-photon absorption (TPA, a list of acronyms can be found at the end of this article) efficiency [1,2,3,4,5], because of relevant applications from photonics to biology, such as microfabrication [6,7,8,9,10,11], micromachines [12], 3D optical data processing and storage [13,14,15,16,17,18], bio-imaging [18,19], and photodynamic therapy [20,21,22,23]. Both experimental and theoretical explorations of the structure–property relationships governing TPA indicated that two critical factors determine the two-photon performances of organic chromophores: the π-conjugation length and the presence of intramolecular charge transfers [3,24,25,26,27,28,29,30,31]. Attention progressively moved from asymmetric Donor–Acceptor systems (D−π−A) to symmetric (A−π−D−π−A or D−π−A−π−D) quadrupolar arrangements and, finally, toward branched molecular structures built from the gathering of either dipolar or quadrupolar chromophores via a common conjugated core [32]. Such design was expected to result at the same time in an increase of active TPA units per molecule and in “through bonds” or “through-space” interactions between these TPA units. If the units behave nearly as independent sub-chromophores, an additive behavior would be observed. Conversely, if some kind of coupling is active between these units, the nonlinear optical (NLO) response would be much higher than what would be expected from the sum of all participating arms. Actually, either an enhancement or a decrease of the TPA activity can be observed depending on the additive, cooperative, or suppressive nature of the inter-branches’ interactions [33,34,35,36]. Besides the all-organic approach, coordination chemistry was rapidly seen as a versatile strategy for designing efficient multipolar two-photon absorbers. The metal ion can act as a powerful template to gather organic TPA chromophores in a predetermined arrangement, while also contributing in intramolecular charge transfers, such as intra-ligand (ILCT), metal-to-ligand (MLCT), ligand-to-metal (LMCT), or ligand-to-ligand (LLCT) charge transfers within the complexes [37,38,39,40].

Among the families of coordination complexes studied in this respect, polypyridyl ruthenium (II) complexes proved to be efficient two-photon activatable models because of their well-established merits, such as: (i) reliable preparations of complexes with predictable structures and tunable photophysical and electrochemical parameters, (ii) a high photostability, and (iii) long-lived triplet states. The most representative studies devoted to the nonlinear optical activity of polypyridyl ruthenium (II) complexes investigated the role of peripheral electron-withdrawing or electron-donating groups in series of homoleptic complexes comprising π-extended bipyridine ligands [34,39,40,41]. It was established by Coe [41,42] and Le Bozec [43,44], for instance, that replacing electron-accepting groups with donor groups strongly enhanced the TPA activity of related Ru(II) polypyridyl complexes. On the other hand, the optical and nonlinear optical properties are strongly influenced by the design of the conjugated π linkers [42]. Limited π-conjugation together with inter-annular twisting within the π-extended bipyridine ligands resulted in relatively modest TPA properties [45]. Recent studies demonstrate the importance of the geometry in the first sphere around the ruthenium core [46], which is consistent with the idea that an octupolar *D*_3_ symmetry should be approached to achieve high cross-section values.

**Scheme 1:**
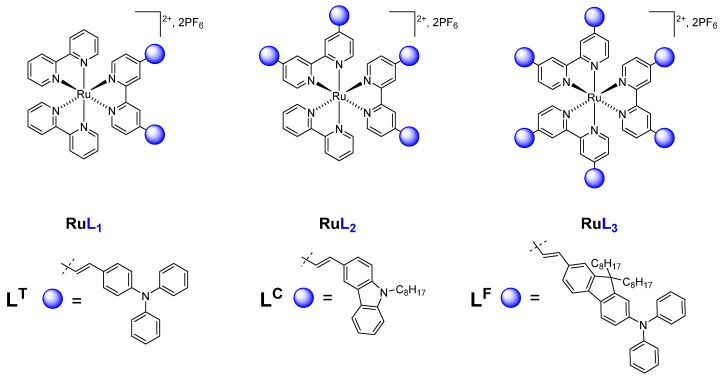
Schematic representation of homoleptic and heteroleptic complexes [RuL_3-n_(bpy)_n_]^2+^ (*n* = 0, 1, 2), L = L^T^, L^C^, L^F^.

We previously [47] described homo- and hetero-leptic complexes bearing π-extended bipyridyl ligands as potentially active TPA sub-chromophores (Scheme 1). The primary objective of this study was to develop series of [RuL_3-n_(bpy)_n_]^2+^ (where bpy represents 2,2′-bipyridine and *n* = 0, 1, 2) complexes to investigate the impact of the number, in each series, and the nature of π-extended ligands around the metal center, from one series to the others, in terms of effective TPA cross-section [48,49]. With this purpose, we designed 2,2′-bipyridine-based ligands where the bipyridine cores were 4,4′-substituted by electron-rich triphenylamine, carbazolyl, and diphenylamino-fluorenyl subunits, through a vinylene linker. The TPA cross-section values, δ, were strongly enhanced when going from the free ligands to the complexes. Interestingly, the measured TPA cross-sections became much higher than the sum of the individual contributions of the bipyridyl ligands. In the present paper, DFT and TD-DFT simulations allow us to rationalize these experimental results: the modification of ligands not only participates to monitor the contributions of both the ligands and metal center to the MOs of the studied complexes, it also affords directionality to the auxiliary metal-to-ligand and ligand-to-ligand CT, promoting either additive, cooperative, or suppressive effects.

## 2. Results

The homoleptic and heteroleptic [RuL_3-n_(bpy)_n_]^2+^ (PF_6_^−^)_2_ (*n* = 0, 1, 2) complexes (Scheme 1) were designed on the basis of 2,2′-bipyridine ligands, L = L^T^, L^C^, L^F^, where the bipyridine cores are 4,4′-substituted by various electron-rich arylamine groups such as triphenylamine (L^T^), carbazolyl (L^C^), and diphenylamino-fluorenyl (L^F^) subunits, through a vinylene linker [47]. The triphenylamine and carbazolyl groups were selected as electron-donating substituents, which would not only modify the D-π-A conjugation, but also tune the architectures in the quadrupolar D-π-A-A-π-D sub-chromophores [27,28]. Similarly, the fluorenyl based ligand (L^F^) was designed because of the utility of fluorenyl units in extending the conjugation length in TPA molecules due to their planar structure [50].

**Figure 1:**
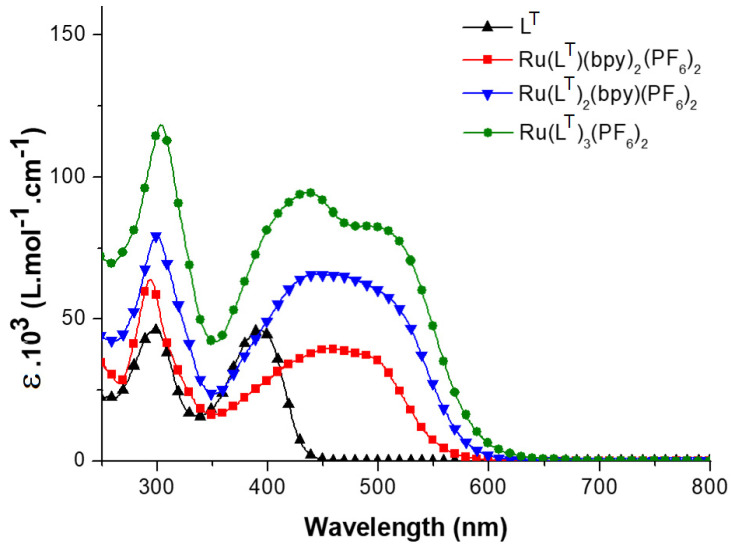
UV-vis absorption spectra of L^T^ and [Ru(L^T^)_3-n_(bpy)_n_]^2+^ (*n* = 0–2) complexes in THF.

The absorption spectra of the ligands along with the corresponding Ru-complexes solutions were carried out (Figure 1 and Supplementary Figures S1 and S2). These data are summarized in Table 1. The ligands display wavelength absorption bands located in the 330–450 nm region, which are both sensitive to the nature of the donor subunit and the conjugation length and were attributed to the ICT transitions. For instance, the bathochromic effect observed from L^C^ to L^T^ (L^T^, λ_max_ = 392 nm; L^C^, λ_max_ = 362 nm) reflects the better donor ability of the triphenylamine moiety compared to the carbazole one [51,52]. On the other hand, the electronic delocalization within the π-conjugated L^F^ bipyridine ligand results in a significant hyperchromic effect (L^T^, λ_max_ = 392 nm, ε = 46.1 × 10^3^ M^−1^.cm^−1^; L^F^, λ_max_ = 395 nm, ε = 76.2 × 10^3^ M^−1^.cm^−1^). These bands are characteristic of intra-ligand charge transfer (ICT) transitions from the peripheral donor amino units to the central acceptor bipyridine moieties [53]. Further bands can be found around 300 nm that were assigned to π-π* transitions within the bipyridine moieties.

The homoleptic [RuL_3_]^2+^ complexes exhibit broad absorption bands in the 500 nm region. In addition to these low-energy absorptions, the complexes exhibit intense bands in the 400–450 nm region. The heteroleptic [RuL_3-n_(bpy)_n_]^2+^ (*n* = 1, 2) complexes also show bands in the 375–460 nm region together with very broad absorption bands in the 470–600 nm region. A hyperchromic effect, relative to the number of extended ligands, is observed for each series. This effect is particularly noteworthy for the RuL^F^ series, for which the conjugation extension within the ligands is the longest. The average positions of the higher-energy absorption maxima ([Ru(L^T^)_3_]^2+^, λ_max_ = 437 nm; [Ru(L^C^)_3_]^2+^, λ_max_ = 420 nm; [Ru(L^F^)_3_]^2+^, λ_max_ = 429 nm) vary according to the nature of the ligands. These variations are weaker, from one series to another, for the lower-energy transitions ([Ru(L^T^)_3_]^2+^, λ_max_ = 504 nm; [Ru(L^C^)_3_]^2+^, λ_max_ = 503 nm; [Ru(L^F^)_3_]^2+^, λ_max_ = 502 nm). The CT band redshift for the ligands in their free form and within the [RuL(bpy)_2_]^2+^ complexes provides some indications on the impact of their coordination to the [Ru(bpy)_2_]^2+^ fragment in each series. The highest energy gap resulting from the coordination (~45 nm, 0.36 eV) is observed for the L^C^ ligand and its complexes, in which the expansion of conjugation is the smallest. Smaller gaps are observed for L^F^ (23 nm, 0.18 eV) and L^T^ (30 nm, 0.23 eV).

All the studied ligands and [RuL_3-n_(bpy)_n_]^2+^ complexes feature emission properties. The ligands are fluorescent, with strong emission in the range 430–500 nm in THF solution (Supplementary Figures S3–S5). Charge transfer characteristics were previously established and support the amplification of ICT transitions when extending the conjugated pathway (L^F^ > L^T^ > L^C^) [47]. The [RuL_3-n_(bpy)_n_]^2+^ are weakly emissive in the range 675–710 nm with luminescence quantum yields (Φ_*em*_) lower than 1 × 10^−2^ in non-degassed THF (Table 1 and Supplementary Figures S3–S5). The peak-to-peak separation between their absorption and emission maxima are about 190 to 205 nm (Stokes Shifts from 0.75 to 0.70 eV). The quantum yields increase by a factor of ~10 in N_2_-saturated THF, indicating a high sensitivity to the presence of oxygen. A redshift in the range of 10–15 nm can be observed from the [RuL(bpy)_2_]^2+^ to the [RuL_3_]^2+^ complexes, in the three series, with the most important shift being observed for the L^C^ series. These emission wavelengths are strongly red-shifted with respect to that of [Ru(bpy)_3_]^2+^ [54], suggesting that the emission could essentially emanate from ^3^MLCT states involving the 4,4′ π-extended bipyridines. On the other hand, the emission quantum yield of [RuL^F^_3-n_(bpy)_n_]^2+^ complexes is observed to be extremely low in comparison to that of the other [RuL_3-n_(bpy)_n_]^2+^ complexes (L^T^, L^C^) at room temperature. These much lower quantum yields for the RuL^F^ series may be indicative of a more efficient partial quenching of the ^3^MLCT state by low-emissive ^3^ILCT (or ^3^LLCT) in this series compared to the other two [55].

**Figure 2:**
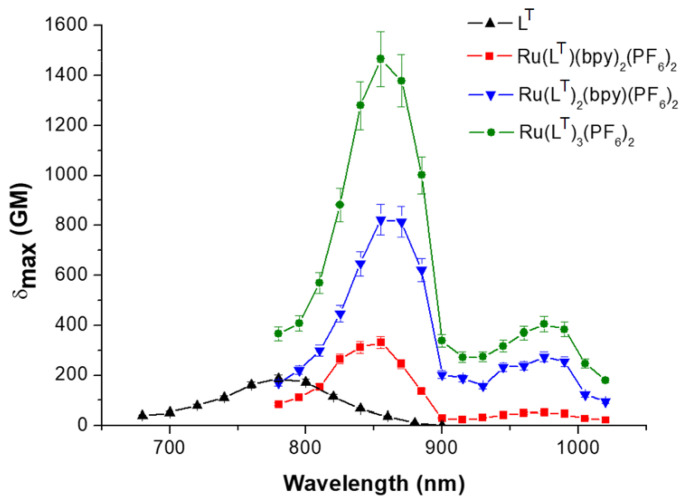
TPA spectra of L^T^ and [Ru(L^T^)_3-n_(bpy)_n_]^2+^ (*n* = 0–2) complexes; THF, c = 1.0 × 10^−5^ mol.L^−1^.

The TPA properties of the ligands and complexes were measured by the two-photon excited fluorescence (TPEF) technique (Supplementary Scheme S1; Figure 2 and Supplementary Figures S6 and S7). Similar emission spectra were observed for each compound with one-photon excitation and multiphoton excitation. The three ligands exhibit similar TPA bands, with maxima absorption wavelengths twice that of their low-energy ICT transitions (L^C^, λ_maxTPA_ = 705 nm; L^T^, λ_maxTPA_ = 780 nm; L^F^, λ_maxTPA_ = 800 nm) and cross-section values around 200 GM (1 GM is 10^−50^ cm^4^ s photon^−1^) (Table 2). Regardless of the series, their complexation induces an amplification of the TPA cross-sections at the respective λ_maxTPA_, by more than a factor of 6 to 8 between the free ligands and their respective homoleptic RuL_3_ complexes. For instance: L^T^, λ_maxTPA_ = 780 nm, δ_max_ = 187 ± 30 GM; [Ru(L^T^)_3_]^2+^, λ_maxTPA_ = 855 nm, δ_max_ = 1465 ± 220 GM (Figure 2). Such a 6- to 8-fold amplification may result from the coordination of the π-stretched ligands to the metal center, which increases the acceptor character of the pyridine rings and from the rigidification of the bipyridine ligands into a planar conformation. On the other hand, the complexation at the metal center obviously results in the multiplication of charge transfers (Supplementary Scheme S2) whose effects are complementary to those of the basic intra-ligand transfers observed in the free ligands.

Only small differences are observed between the TPA spectra of the [RuL_3-n_(bpy)_n_]^2+^ (*n* = 0–2) complexes in THF when the general shape and wavelengths are considered. These spectra feature intense bands in two perfectly distinct regions corresponding to half the energy of the OPA transitions with a marked CT character, i.e., from 350 to 600 nm (Figure 2 and Supplementary Figures S6 and S7). The similarity in the spectral shape of the TPA spectra and the CT bands suggests that the first excited states are both one- and two-photon allowed, which corresponds to the classical behavior of dipolar or multipolar structures, with a reduced symmetry. Moderate- to low-intensity two-photon absorption bands are observed for all series at 900–1050 nm wavelengths that could correspond to half the energy of the OPA transitions located at 450–600 nm. More intense TPA bands are visible at higher energies (775–900 nm), corresponding to roughly twice the OPA transitions located at 350–400 nm (Figure 2, Supplementary Figures S6 and S7, Table 2). The relative intensities of the two TPA bands all along the three series vary with the number of π-extended ligands. They also depend on the nature of these ligands: The highest ratio δ_max LE_/δ_max HE_ (LE: low energy, HE: high energy) is observed for the TPEF spectra of the L^F^ series, and the lowest ratio for the L^C^ series. This is similar with what can be observed in OPA.

While the λ_maxTPA_ vary only slightly within each series, a significant increase of the δ_max_ values, corresponding to maxima located in the 775–900 nm window, can be observed when comparing the variations from [Ru(bpy)_3_]^2+^ to the [RuL_3-n_(bpy)_n_]^2+^ complexes (Table 2). On the other hand, the highest difference between the experimental δ_max_ values for the free ligands and the corresponding [RuL(bpy)_2_]^2+^ complexes is observed for [RuL^F^(bpy)_2_]^2+^ (approximately 390 GM), and the lowest gap for [RuL^T^(bpy)_2_]^2+^ (approximately 125 GM).

One would expect the two-photon cross-section in each series to follow a 1:2:3 ratio, from the [RuL(bpy)_2_]^2+^ to the [RuL_3_]^2+^ complexes, provided that these multi-branched complexes consist of non-interacting units. However, if we compare the δ_max_ values of the [RuL_3_]^2+^ complexes, the lowest δ_max_ values correspond to [Ru(L^F^)_3_]^2+^ (λ_maxTPA_ = 870 nm, δ_max_ = 1315 ± 200 GM), and the highest to [Ru(L^T^)_3_]^2+^ (λ_maxTPA_ = 855 nm, δ_max_ = 1465 ± 220 GM). This observation is counterintuitive since the δ_max_ values of the complexes are respectively the highest [Ru(L^F^)(bpy)_2_]^2+^ (δ_max_ = 596 ± 120 GM) and the lowest [Ru(L^T^)(bpy)_2_]^2+^ (δ_max_ = 311 ± 60 GM) (Table 2). To more accurately compare the TPA cross-section values for the three series of complexes, the δ_max_ values were normalized by N_*eff*_^2^, by applying the method proposed by Kuzyk et al. [48,49], where N_*eff*_ represents the effective number of π-electrons contributing to the nonlinear response of the chromophores (Table 2). N_*eff*_ can be determined by geometrically weighting the number of electrons in each conjugated path of the molecular system (see the Supplementary Information).

**Figure 3:**
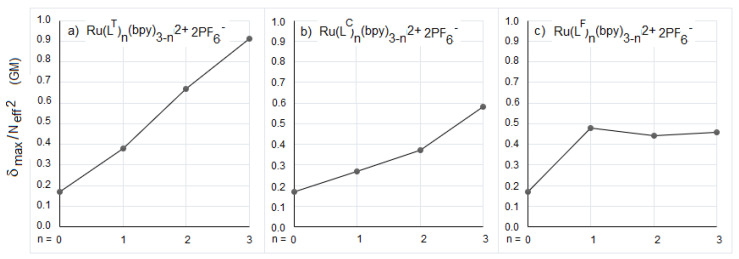
(**a**–**c**). Evolution of intrinsic TPA cross-sections as a function of the number of ligands, L^T^ (**a**), L^C^ (**b**), and L^F^ (**c**), in [RuL_3-n_(bpy)_n_]^2+^ complexes. Ru(bpy)_3_^2+^ acts as a reference (*n* = 0) [46].

The intrinsic TPA cross-sections, measured at the respective λ_maxTPA_ of the complexes, are reported in Figure 3a–c as a function of the number of ligands L^T^, L^C^, and L^F^ around the metal. For the L^F^ series, the intrinsic TPA reaches a plateau as soon as the first ligand is introduced, that corresponds to a notable enhancement of the intrinsic TPA cross-sections for [RuL^F^(bpy)_2_]^2+^ compared to [Ru(bpy)_3_]^2+^ (Figure 3c). Meanwhile, the intrinsic cross-sections for the three [Ru(L^F^)_3-n_(bpy)_n_]^2+^ complexes (*n* = 0–2) are perfectly similar (approximately 0.46 GM). These slight variations suggest that the progressive addition of L^F^ ligands around the metal does not introduce any effect other than additive, for this series. The variations of the intrinsic cross-sections for the [RuL_3-n_(bpy)_n_]^2+^ complexes (L = L^C^ or L^T^) clearly differ from that for the RuL^F^ series. A quasi-linear enhancement of the intrinsic TPA values related to the number of ligands is observed for these two series (Figure 3a,b). These observations not only indicate that the TPA cross-section values of the RuL^C^ and RuL^T^ series of complexes are sensitive to the increase in the number of 4,4′ π-extended bipyridines, but suggest that additional contributions allow for a cooperative effect in these series, that could result from interbranch electronic coupling or from an extent of the charge transfer path among the different branches.

### 2.1. Computational Results

#### 2.1.1. Geometry Optimization

The [Ru(L)_3-n_(bpy)_n_]^2+^ (*n* = 0, 2) complexes at their ground state (S_0_) exhibit slightly distorted octahedral geometries (Supplementary Table S1). However, the Ru-N bond lengths do not significantly differ from one series of complexes to another one, which means that the structural modification of the π-conjugated ligands induces minor effects on the ground state geometry of these complexes in the immediate vicinity of the metal center. On the other hand, a quite negligible effect is observed on the main structural features of the [Ru(L)_3-n_(bpy)_n_]^2+^ (*n* = 0, 2) complexes when considering the effect of heterogenization in a series. For example, average bond lengths of 2.060 Å for Ru-N relative to the L-ligands were found for the homogeneous (*n* = 0) and heterogeneous (*n* = 2) complexes, respectively. These values do not significantly differ from the average bond lengths of 2.059 Å calculated for Ru-N relative to the bpy-ligands in the heterogeneous complexes, in the three series. The trans N-Ru-N bond angles for the coordinated L- and bpy-ligands are in the range from 173.18 to 174.26° overall. Thus, they slightly deviate from the theoretical value for a perfect octahedral geometry around the ruthenium atom. The optimized geometries depart slightly from a perfect octahedral geometry, with the angles between two Ru-L bonds being equal to ca. 88° instead of the ideal 90° value for the three different RuL3 complexes. The ligands exhibit different lengths, i.e., 14.7, 15.47, and 18.7 Å, respectively, for the L^T^, L^C^, and L^F^ ligands in the complexes. One can notice that these distortions from the octahedral geometry do not significantly vary from heterogeneous to homogeneous complexes, from one series to another one, or within a series. Finally, in all the optimized structures, the substituting styryl-groups are coplanar with the bipyridyl ligand to which they are attached. In a more comprehensive perspective, the overall similarity of the geometrical parameters suggests that the local structure in the complexes, especially the close environment of the Ru, is not significantly perturbed by the nature and functionalization the bpy-type ligand.

#### 2.1.2. Molecular Orbitals

The Highest Occupied Molecular Orbitals (HOMO) and the Lowest Unoccupied MOs (LUMO) of the ligands L^T^, L^C^, and L^F^ are shown in Supplementary Figure S8. The HOMOs are mainly localized on the donating peripheral groups, and cover the styryl fragments to some extent. For L^F^, the pyridine rings do not participate to the HOMO and HOMO-1, contrary to L^C^ and L^T^. The LUMO orbitals of L^C^ and L^F^ are localized mostly on the pyridine rings, and diffuse symmetrically into the carbazolyl or fluorenyl rings. For their part, the LUMO and LUMO+1 of L^T^ show a mirrored localization on the pyridine rings and one branch of the styryl-diphenylamine system each. For all three structures, the contributions to the first excited state are mostly HOMO-1→LUMO and HOMO→LUMO+1. One can notice that the energy differences are small (<0.03 eV) between HOMO and HOMO-1, and relatively small between the LUMO and LUMO+1 (respectively, L^T^ 0.11 eV, L^C^ 0.21 eV, L^F^ 0.16 eV) (Supplementary Table S2). The smallest HOMO–LUMO gap is observed for L^F^ (3.15 eV), which combines the strongest donor (−5.19 eV) and the best acceptor (−2.04 eV) systems. L^C^ has the lowest donor (−5.65 eV) and the largest HOMO–LUMO gap (3.68 eV), a value that is close to that for L^T^ (3.52 eV) which has the lowest acceptor (−1.77 eV).

**Figure 4:**
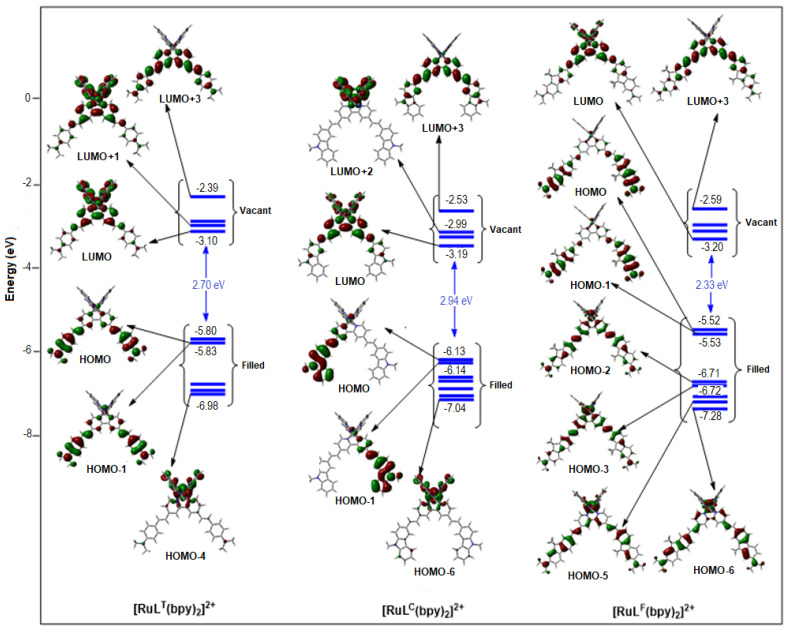
PBE0-GD3BJ/LANL2DZP. Relevant frontier MOs with their energies (in eV) of [RuL^T^(bpy)_2_]^2+^ (**left**), [RuL^C^(bpy)_2_]^2+^ (**middle**), and [RuL^F^(bpy)_2_]^2+^ (**right**) calculated at the PBE0-GD3BJ/6-31G* level, in solution. An iso-value of 0.03 au was used.

The MOs of the [Ru(L)_3-n_(bpy)_n_]^2+^ (*n* = 0, 2), which have important contributions to the electronic transitions during one- and two-photon excitations, are collected in Figure 4 ([RuL(bpy)_2_]^2+^ complexes) and Supplementary Figures S9–S11 ([Ru(L)_3_]^2+^ complexes).

The DFT calculations indicate that the nature of the substituents of the vinyl-bipyridine L-ligands strongly alters the composition of the frontier orbitals of the complexes. One can notice how the calculated contributions of the metal (4*d*-Ru) to the HOMO and HOMO-1 orbitals confirm this statement (Table 3 and Supplementary Tables S3–S5). For instance, the metal contributions are respectively 18% and 9% for [Ru(L^T^)(bpy)_2_]^2+^ (Supplementary Table S3) but only 5% and 4% for [Ru(L^C^)(bpy)_2_]^2+^ (Supplementary Table S4). For [Ru(L^F^)(bpy)_2_]^2+^, these metal contributions drop to 1% for both the HOMO and HOMO-1, where the main contribution corresponds to the vinyl-substituted bipyridine ligands (Supplementary Table S5). In this case, the 4*d*-Ru contribution involves HOMO-2, at a very negative energy level (−6.71 eV vs. −5.53 eV for HOMO-1). As previously observed for the free ligand L^F^, the pyridine rings marginally take part in the HOMO and HOMO-1 of [Ru(L^F^)(bpy)_2_]^2+^. The electron densities are predominantly distributed on the fluorenyl-diphenyl amine fragments of the ligands in the complex. This is a specific feature of this series, compared to the RuL^T^ and RuL^C^ series, in which both the metal center and the bipyridyl fragments participate in the electron densities of the HOMO and HOMO-1. [Ru(L^F^)_3_]^2+^ also differs from [Ru(L^T^)_3_]^2+^ and [Ru(L^C^)_3_]^2+^ for the metal contribution to their respective frontier HOMO orbitals. While at around 10% for the last two, it is practically absent in the frontier orbitals of [Ru(L^F^)_3_]^2+^, for which it is necessary to reach the HOMO-6 level to observe a significant metallic contribution (44%).

The LUMO and LUMO+1 of [Ru(L^C^)(bpy)_2_]^2+^ and [Ru(L^F^)(bpy)_2_]^2+^ show great similarities. The vinyl-substituted bipyridines as well as the unsubstituted bipyridine ligands contribute in a shared manner to the LUMO and LUMO+1 of these complexes, with percentage weights of 78% and 83% each. This situation is in stark contrast to the one faced by [Ru(L^T^)(bpy)_2_]^2+^, where the contribution of the unsubstituted bpy-ligands achieves weights of around 77% for both the LUMOs and LUMO+1, while the weight of the vinyl-substituted bipyridines is no more than 8%. This observation reflects the lower electron-withdrawing character of L^T^ (LUMO energy level: −1.77 eV compared to −1.97 eV (L^C^) and −2.04 eV (L^F^)). One can also notice that the metal contribution to the LUMO and LUMO+1 is never zero (2% to 4% weight for [Ru(L^C^)(bpy)_2_]^2+^ or [Ru(L^F^)(bpy)_2_]^2+^, but is high, 7% to 20%, for [Ru(L^T^)(bpy)_2_]^2+^).

#### 2.1.3. TD-DFT Calculations of the Electronic Absorption Spectra

A semi-quantitative agreement is found between the calculated transition energies and the experimentally observed one-photon absorption spectra, even if the intensities are not qualitatively reproduced (Supplementary Tables S6–S11). PBE0 simulations are closer to the experimental observations for the RuL^T^ and RuL^C^ series, whereas CAM-B3LYP ones appear more appropriate for the RuL^F^ series. This is likely due to the fact that the charge transfer following the S0 to S1 excitation is more important in the case of the ligand L^F^ and the related complexes. In many respects, the calculated absorption spectra of the homogeneous [Ru(L)_3_]^2+^ complexes look similar to that of the heterogeneous [Ru(L)(bpy)_2_]^2+^ complexes. In both cases, two bands are identified that coincide well with the experimental spectra from 300 to 800 nm. For instance, two maxima are calculated around 400 and 500 nm, respectively, for the [Ru(L^C^)(bpy)_2_]^2+^ and [Ru(L^C^)_3_]^2+^complexes (Supplementary Table S7). For [Ru(L^C^)(bpy)_2_]^2+^, the lowest energy absorption manifold is a combination of intra-ligand transitions within the carbazolyl-substituted ligand L^C^ (from HOMO-1 and HOMO to LUMO) and of MLCT transitions from Ru *d* orbitals to ligand L^C^. The higher-energy absorption also corresponds to intra-ligand charge transfers directed to the carbazolyl-substituted ligand (LUMO+3), together with MLCT transitions from Ru *d* orbitals (HOMO-6) to the unsubstituted bpy-ligands (LUMO+2). For [Ru(L^C^)_3_]^2+^, the low-energy (LE) absorption band (λ_max_ = 503 nm) is composed of a combination of excitations from HOMO, HOMO-1, and HOMO-3 to the LUMO, LUMO+1, and LUMO+2. The higher-energy (HE) absorption manifold is composed by two excitations from HOMO-7 to LUMO and HOMO-2 to LUMO+1. Both transitions can be designated as LLCT/ILCT transitions with a MLCT contribution, a main difference being the metallic contribution to these transitions (LE: HOMO: Ru(%) = 12%, HOMO-1: 10%, and HOMO-3: 21%. HE: HOMO-7: 8%, HOMO-2: 1%).

The RuL^T^ series contrasts with the RuL^C^ series due to the fact that the LUMO and LUMO+1 are mainly located on the unsubstituted bpy for [Ru(L^T^)(bpy)_2_]^2+^ (Supplementary Table S3). For this complex, the lowest energy absorption is a combination of transitions from HOMO and HOMO-1 to LUMO and LUMO+1 and is characterized by MLCT and LLCT to the unsubstituted bpy (Supplementary Table S10). The highest energy absorption corresponds to HOMO-1 to LUMO+3 and HOMO-4 to LUMO transitions, that is a combination of MLCT transitions (with a noticeable contribution of the metal: Ru(%) = 9% in HOMO-1 and 66% in HOMO-3) to the unsubstituted bpy and ILCT within L^T^. For [Ru(L^T^)_3_]^2+^, the lowest energy band (λ_max_ = 525 nm) consists of mixed MLCT, ILCT, and LLCT transitions from HOMO, HOMO-1, and HOMO-2 (metallic weights: 11%, 11%, and 10%) to LUMO and LUMO+1. The highest energy absorption (λ_max_ = 447 nm) is a combination of MLCT and ILCT transitions mixed with LLCT transitions from HOMO-1 and HOMO-2 to LUMO+3.

[Ru(L^F^)(bpy)_2_]^2+^ and [Ru(L^F^)_3_]^2+^ differ from their homologues due to the absence of metallic contribution in the higher-energy HOMO and HOMO-1 (Supplementary Table S5): in these systems, the intra-ligand charge transfer (ILCT) from the diphenylamino groups to the pyridine moieties predominates over the other (MLCT or LLCT) charge transfers. For [Ru(L^F^)(bpy)_2_]^2+^, the LE absorption band, for which the calculated maximum is located at 430 nm, corresponds to a combination of multiple transitions involving HOMO, HOMO-1, HOMO-2, and HOMO-3 to LUMO and LUMO+3, in which the unsubstituted bpy-ligand is under-represented (Supplementary Tables S5 and S11). The higher-energy band (λ_max_= 335 nm) has a marked ILCT character associated with a ML^F^CT contribution (supported by a noticeable contribution of the metal: Ru(%) = 66% in the HOMO-4). For [Ru(L^F^)_3_]^2+^, the lowest-energy band is a mixed character ILCT over each of the three coordinated ligands, with some MLCT character. The highest-energy band presents a ILCT character with a MLCT contribution (HOMO-6: (Ru(%) = 44%) to L, Supplementary Table S5), which is distributed over all the L^F^ ligands.

## 3. Discussion

Although the OPA- and TPA-allowed transitions are not necessarily the same, we assume that the description of the MOs involved in these two absorption modes can be used to draw up an overall picture. This assertion is also supported by the similarity in the spectral shape of the TPA spectra and the CT bands, which suggests that the first excited states are both one- and two-photon allowed. Interestingly, the above computational results provide an explanation for the presence of the two experimentally distinct two-photon absorption active windows (the first one at low energy (LE) from 950 to 1000 nm, and the second at higher energy (HE) from 700 to 850 nm) and makes it possible to discuss trends regarding their nature. It appears that these active windows essentially differ in the composition of the frontier HOMOs of distinct energy levels: upper HOMO levels are the main ones involved in LE transitions while the HE transitions also involve deeper HOMOs. The LUMO levels are of the same nature, essentially LUMO to LUMO+2 or LUMO+3. Regarding the issue of the nature of the reached excited states, it is worth noting that there were numerous excited states and not a single one lay in a narrow energy range around twice the TPA energy value.

**Figure 5:**
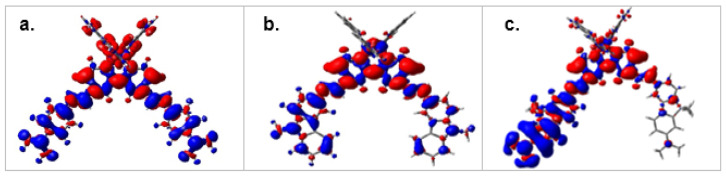
(**a**–**c**) PBE0-GD3BJ/LANL2DZP density differences plots (Dρ(r) = ρ^S1^(r) − ρ^S0^(r)) of [RuL (bpy)_2_]^2+^ complexes, (**a**) L = L^T^, (**b**) L = L^C^, and (**c**) L = L^F^) between S_1_ and S_0_ states (red = increase, blue = decrease of electron density; iso-value 0.03 au).

The above computational studies confirm that ILCTs are the keystone of the CT-based absorption bands in the studied complexes (Figure 5 and Supplementary Figure S12). These major contributions are affected by MLCT and LLCT, whose impact depends on the number and the nature of the substituted ligands in the complexes. Any contribution that extends the conjugation pathway from an π-extended ligand would be considered cooperative, while any effect that favors intra-ligand localization would be considered suppressive. Accordingly, LLCTs are expected to promote TPA amplification, since they contribute to an enhanced π-electron delocalization beyond a single ligand. Moreover, their overall impact is expected to increase with the number of π-stretched ligands, as with ILCTs. The impact of MLCTs is more equivocal and is definitely conditioned by the contribution of the unsubstituted bpy- or L-ligands to the involved LUMOs. It can be assumed that the MLCTs are defined by a suppressive effect when oriented towards the π-extended ligands, but a contributing effect if oriented towards an unsubstituted bipyridyl ligand.

From this perspective, the [Ru(L^F^)_3-n_(bpy)_n_]^2+^ series differs from the other two series by a small metal contribution to the H0 to H-2 and L0 to L+3 MOs that are key components for the ground and singlet (and even triplet) excited states. These statements are consistent with the weaker emission quantum yields for the RuL^F^ complexes compared to the other series, that can be attributed to a partial quenching of ^3^MLCT by ^3^ILCT. This is consistent with the weak variations of experimental values of the intrinsic cross-sections, which appear to be mainly additive within this series (Figure 3c). Looking at the MOs involved in the HE transitions for [Ru(L^F^)(bpy)_2_]^2+^, a strong metallic contribution to the HOMOs and a major L^F^ contribution to the LUMOs are observed (Supplementary Table S5), that could be favorable to a single-branch IL dipolar CT in this complex (Figure 5c), with the MLCT and ILCT contributions being essentially opposite. Conversely, the higher δ_max LE_/δ_max HE_ (LE: low energy, HE: high energy) TPA ratio observed for the RuL^F^ series could also be related to a minimized metallic participation for the higher0energy HOMOs, resulting in LLCT or MLCT with moderate to weak effects. The additive increase of the cross-section values for the RuL^F^ series thus results in prominent dipolar ILCTs in this series, to be correlated with the longer conjugated path length for the L^F^ ligands.

A simple additive effect that would have the ILCTs as its unique origin cannot account for the evolution of the cross-section values reported for the RuL^T^ and RuL^C^ series. We assume that their increase from one complex to another, within each of these series, results from prominent ILCT summative effects together with MLCT and LLCT auxiliary contributions, with the metal center acting beyond a simple aggregator. This specific role will be especially important if the metallic contributions to the HOMOs and LUMOs involved in the transitions giving rise to TPA are significant, as observed for the L^T^ and L^C^ series.

The high metallic contribution at the LUMO to LUMO+3 levels (up to 20% in [Ru(L^T^)(bpy)_2_]^2+^) can promote MLCT and LLCT contributions beyond the metallic center. The MLCT and LLCT contributions are mainly oriented from M (or L^T^) to the unsubstituted bpy-ligands. This configuration is particularly suitable for an extension of the conjugation in this complex, as can be seen in Figure 5a, where the density differences plot (Dρ(r)= ρ^S1^(r) − ρ^S0^(r)) between S_1_ and S_0_ states encompasses the entire conjugated system. Similarly, the LLCT contributions beyond the metallic center are expected to spread over all extended L^T^ ligands in [Ru(L^T^)_3_]^2+^. The situation is quite similar for the RuL^C^ series, where the metallic contribution to the HOMOs and the nature of the LUMOs involved in the HE transitions support an extension of conjugation to the whole system (Figure 5b), with the main differences being in the contributions of bpy and L^C^ to the lower-energy LUMOs in [Ru(L^C^)(bpy)_2_]^2+^.

The low-energy TPA excited states of the complexes are mainly of ILCT and LLCT character, with these excitations involving HOMO and HOMO-1 to LUMO and LUMO+1 transitions, similar to the OPA leading to the S1 excited state. Experimentally, it has been seen that the cross-section is much higher in the case of the complexes with the L^F^ ligand than for L^T^ and L^C^ complexes. This is mainly due to the highest ILCT charge transfer in the case of the L^F^ complexes (Supplementary Figure S12) related to the longest conjugated pathway and the greater donating strength of the L^F^ ligand. In order to study this point more thoroughly, we computed the dipole moments in the vertical excited S1 state of all compounds (Supplementary Table S12). A high difference between the S0 ground state and S1 dipole moment should be associated with a high TPA response. Indeed, the dipole moments’ differences between the vertical S1 and the S0 states are 11.38, 8.30, and 4.27 Debye, respectively, for [Ru(L^F^)_3_]^2+^, [Ru(L^T^)_3_]^2+^, and [Ru(L^C^)_3_]^2+^, correlating well with the LE TPA cross-sections of these complexes, i.e., 681, 405, and 173 GM.

## 4. Materials and Methods

### 4.1. Synthesis and Photochemical Measurements

The synthetic details for the bipyridyl ligands L^T^, L^C^, and L^F^, and related homoleptic and heteroleptic [RuL_3-n_(bpy)_n_]^2+^ (*n* = 0, 1, 2) complexes were described previously [47] (see also Supplementary Information). UV-vis absorption spectra were recorded using a UVIKON 9413 (Secomam SA—Alès, France) or Biotek Instruments XS spectrophotometer (Secomam SA—Alès, France) using quartz cuvettes of a 1 cm path length. Steady-state luminescence spectra were measured using a Jobin Yvon FluoroMax-4 (Horiba France Sas—Longjumeau, France) or Tau-3 spectrofluorimeter (Horiba France Sas—Longjumeau, France). The two-photon absorption (TPA) measurements were performed with femtosecond mode-locked laser pulse using a Ti:Sapphire laser (Coherent, Chameleon Ultra II: pulse duration: ~140 fs, repetition rate: 80 MHz, wavelength range: 680–1040 nm) (Coherent Inc., Santa Clara, CA, USA) [47].

### 4.2. Theoretical Computations

The DFT calculations have been performed using the Gaussian 09 suite of programs [49]. Calculations were performed for the ligands L^T^, L^C^, and L^F^ and the corresponding [Ru(L)_3-n_(bpy)_n_]^2+^ complexes with *n* = 0 and *n* = 2. Methyl groups were used instead of phenyl ones in L^T^ or L^F^ and in the corresponding complexes, and instead of the n-octyl chains (L^C^ and L^F^). The structures were fully optimized using the PBE1PBE [56,57,58] functional, and including GD3BJ [59,60] empirical dispersion corrections and the Lanl2DZ basis set augmented with polarization functions for all atoms, except hydrogen. The nature of the stationary points after optimization was checked by calculations of the harmonic vibrational frequencies to ensure that genuine minima were obtained. Time-dependent density functional theory (TD-DFT) calculations were performed employing the two functionals, PBE1PBE and CAM-B3LYP [61], using the previously optimized geometries. During all the calculations, the solvent effects, in our case THF, were taken into account by means of the Polarizable Continuum Model (PCM) [62]. Molecular orbitals and theoretical absorption spectra were plotted using the GaussView program(Semichem Inc., Shawnee, KS, USA) [63].

## 5. Conclusions

The analysis presented here is a step forward to promote two-photon absorption properties of homo- and hetero-leptic [RuL_3-n_(bpy)_n_]^2+^ polypyridine ruthenium complexes. The resulting model, supported by theoretical calculations, highlights how much the one-photon absorption of these complexes results from a mix of ILCT, MLCT, and LLCT transitions, arising from dipolar transitions rather than octupolar ones. Even if a parallel between the TPA and OPA may be limited because of the lack of knowledge of authorized TPA transitions in the studied complexes, one conceives that the above ILCT, MLCT, and LLCT charge transfers are also essential components of the two-photon response of the [RuL_3-n_(bpy)_n_]^2+^ complexes. The concomitance of the wavelength windows involved in both OPA and TPA supports this hypothesis.

Extending the conjugated system or enhancing the donor effect of peripheral units of the π-extended L-ligands favors ILCTs as the main contributors to TPA transitions, corresponding to half the energy of the OPA transitions with a marked CT character. Meanwhile, our studies demonstrated that the number of π-extended ligands around the metal does not fully account for the TPA response of the complexes, which is not necessarily purely additive. We observed that the relative metallic weight in the frontier orbitals (HOMOs, and to a lesser extent LUMOs) of the complexes impacts on these MLCT and LLCT contributions and may help promote an extension of conjugation on the whole systems, and thus the participation of an increased number of π-electrons participating to the process. Either suppressive or cooperative effects can arise from the resulting MLCT and LLCT contributions, depending on the respective orientations of both ILCT, MLCT, and LLCTs. Therefore, introducing ligands, which allow the MLCT and LLCT charge transfers to be oriented concomitantly with the ILCT ones, merges as a new option that would favor the design of heteroleptic complexes with strong two-photon absorption.

## Figures and Tables

**Table 1:** Main one-photon absorption and emission characteristics of the ligands (L = L^T^, L^C^, L^F^) and related [RuL_3-n_(bpy)_n_]^2+^ complexes (*n* = 0–2) in THF.

	λ_abs_ (nm)	ε.10^3^ (L·mol^−1^·cm^−1^)	λ_em_ (nm)	Φ_em_ (N_2_ atm.)
L^T^	299, 392	46, 46	481	0.80
[Ru(L^T^)(bpy)_2_]^2+^	294, 455, 489	64, 40, 37	695	0.0013 (0.014)
[Ru(L^T^)_2_(bpy)]^2+^	300, 445, 505	79, 66, 59	700	0.0021 (0.031)
[Ru(L^T^)_3_]^2+^	303, 437, 504	119, 94, 82	705	0.0032 (0.041)
L^C^	300, 362	44, 51	433	0.11
[Ru(L^C^)(bpy)_2_]^2+^	294, 415, 469	71, 34, 30	675	0.0034 (0.017)
[Ru(L^C^)_2_(bpy)]^2+^	300, 415, 494	100, 80, 55	685	0.0057 (0.047)
[Ru(L^C^)_3_]^2+^	303, 420, 503	147, 141, 86	695	0.0063 (0.056)
L^F^	309, 395	57, 76	498	0.78
[Ru(L^F^)(bpy)_2_]^2+^	294, 450, 493	101, 66, 62	695	0.0012 (0.017)
[Ru(L^F^)_2_(bpy)]^2+^	300, 426, 507	110, 100, 88	700	0.0018 (0.017)
[Ru(L^F^)_3_]^2+^	309, 429, 502	146, 139, 122	705	0.0028 (0.029)

**Table 2:** Main one-photon (OPA) and two-photon (TPA) absorption characteristics of the ligands (L = L^T^, L^C^, L^F^) and related [RuL_3-n_(bpy)_n_]^2+^ complexes (*n* = 0–2).

	λ_abs_ (nm) OPA	λ_abs_ (nm) TPA	δ_max_ (GM)	N_*eff*_	δ_max_/N_*eff*_^2^ (GM)
L^T^	299, 392	780	187	23.1	0.35
[Ru(L^T^)(bpy)_2_]^2+^	294, 455, 489	855	311	28.6	0.38
[Ru(L^T^)_2_(bpy)]^2+^	300, 445, 505	855	822	35	0.67
[Ru(L^T^)_3_]^2+^	303, 437, 504	855	1465	40.1	0.91
L^C^	300, 362	705	200	28.3	0.25
[Ru(L^C^)(bpy)_2_]^2+^	294, 415, 469	795	395	38.3	0.27
[Ru(L^C^)_2_(bpy)]^2+^	300, 415, 494	840	655	42.1	0.37
[Ru(L^C^)_3_]^2+^	303, 420, 503	855	1381	48.8	0.58
L^F^	309, 395	800	208	30.7	0.22
[Ru(L^F^)(bpy)_2_]^2+^	294, 450, 493	855	596	35.2	0.48
[Ru(L^F^)_2_(bpy)]^2+^	300, 426, 507	870	889	44.9	0.44
[Ru(L^F^)_3_]^2+^	309, 429, 502	870	1315	53.5	0.46

**Table 3:** PBE0-GD3BJ/LANL2DZP calculated energies and metal contribution in frontier MOs.

	HOMO-2	HOMO-1	HOMO	LUMO	LUMO+1	LUMO+2
[Ru(L^T^)(bpy)_2_]^2+^ (% Ru)	−6.81 eV (66)	−5.83 eV (9)	−5.80 eV (18)	−3.10 eV (7)	−2.98 eV (20)	−2.95 eV (11)
Ru(L^C^)(bpy)_2_]^2+^ (% Ru)	−6.71 eV (7)	−6.14 eV (4)	−6.13 eV (5)	−3.19 eV (2)	−3.04 eV (4)	−2.99 eV (6)
[Ru(L^F^)(bpy)_2_]^2+^ (% Ru)	−6.71 eV (43)	−5.53 eV (1)	−5.52 eV (1)	−3.20 eV (2)	−3.04 eV (4)	−2.98 eV (6)
[Ru(L^T^)_3_]^2+^ (% Ru)	−5.68 eV (10)	−5.67 eV (11)	−5.67 eV (11)	−2.92 eV (1)	−2.78 eV (6)	−2.72 eV (5)
[Ru(L^C^)_3_]^2+^ (% Ru)	−6.14 eV (1)	−6.07 eV (10)	−6.04 eV (12)	−3.20 eV (1)	−3.07 eV (6)	−3.07 eV (6)
[Ru(L^F^)_3_]^2+^ (% Ru)	−5.51 eV (0)	−5.49 eV (0)	−5.48 eV (0)	−3.16 eV (0)	−3.04 eV (6)	−3.03 eV (6)

## Data Availability

Not applicable.

## Supplementary Materials

The following supporting information can be downloaded online. Synthesis and main characterizations, absorption, emission, and TPA spectra of the complexes, energy diagram of the frontier MOs of the ligands and some of the complexes, and finally TD-DFT spectral calculations are reported. The following supporting information can be downloaded online: Scheme S1: Simplified Jablonski diagram for two-photon excitations in [Ru(bpy)_3_]^2+^-like complexes. Scheme S2: Main charge transfers upon excitation within Ru(L^T^)_3_ as a model of complexes containing extended π-system ligands. Figure S1: UV-vis absorption spectra of L^C^ and [Ru(L^C^)_3-n_(bpy)_n_]^2+^ (*n* = 0–2) complexes; Figure S2: UV-vis absorption spectra of L^F^ and [Ru(L^F^)_3-n_(bpy)_n_]^2+^ (*n* = 0–2) complexes; Figure S3: Emission spectra of L^T^ and [Ru(L^T^)_3-n_(bpy)_n_]^2+^ (*n* = 0–2) complexes; Figure S4: Emission spectra of L^C^ and [Ru(L^C^)_3-n_(bpy)_n_]^2+^ (*n* = 0–2) complexes; Figure S5: Emission spectra of L^F^ and [Ru(L^F^)_3-n_(bpy)_n_]^2+^ (*n* = 0–2) complexes; Figure S6: TPA spectra of L^C^ and [Ru(L^C^)_3-n_(bpy)_n_]^2+^ (*n* = 0–2) complexes; Figure S7: TPA spectra of L^F^ and [Ru(L^F^)_3-n_(bpy)_n_]^2+^ (*n* = 0–2) complexes; Page 6: Examples of calculation of the effective number of electrons involved in two-photon transitions. Figure S8: Energy diagram of the frontier MOs of the ligands. Figure S9: main MOs of [Ru(L^T^)_3_]^2+^. Figure S10: Main MOs of [Ru(L^C^)_3_]^2+^. Figure S11: Main MOs of [Ru(L^F^)_3_]^2+^. Figure S12: Density differences plots (Dρ(r)= ρ^S1^(r) − ρ^S0^(r)) of of L and related [RuL_3-n_(bpy)_n_]^2+^ complexes (*n* = 0–2), between S_1_–S_0_ states (red=increase, blue=decrease of electron density; isovalue 0.03 au).Table S1: Main structural characteristics of L and related [RuL_3-n_(bpy)_n_]^2+^ complexes. Table S2: Energies of the frontier MOs of bipyridines L = L^T^, L^C^, L^F^. Table S3: Energies and percentage contributions of the frontier MOs of [RuL^T^_3-n_(bpy)_n_]^2+^ complexes. Table S4: Energies and percentage contributions of the frontier MOs of [RuL^C^_3-n_(bpy)_n_]^2+^ complexes. Table S5: Energies and percentage contributions of the frontier MOs of [RuL^F^_3-n_(bpy)_n_]^2+^ complexes. Table S6: PBE0-GD3BJ and CAM-B3LYP-GD3BJ simulated UV-visible spectra of L^T^ and related [RuL^T^_3-n_(bpy)_n_]^2+^ complexes. Table S7: PBE0-GD3BJ/Lanl2DZP and CAM-B3LYP-GD3BJ/Lanl2DZP simulated UV-visible spectra of L^C^ and related [RuL^C^_3-n_(bpy)_n_]^2+^ complexes. Table S8: PBE0-GD3BJ/Lanl2DZP and CAM-B3LYP-GD3BJ/Lanl2DZP simulated UV-visible spectra of L^F^ and related [RuL^F^_3-n_(bpy)_n_]^2+^ complexes. Table S9: PBE0-GD3BJ and CAM-B3LYP-GD3BJ calculated maximum absorption wavelengths (λ, nm) and oscillator strengths (*f*) in THF for L^T^ and related [RuL^T^_3-n_(bpy)_n_]^2+^ complexes. Table S10: PBE0-GD3BJ and CAM-B3LYP-GD3BJ calculated maximum absorption wavelengths (λ, nm) and oscillator strengths (*f*) in THF for L^C^ and related [RuL^C^_3-n_(bpy)_n_]^2+^ complexes. Table S11: PBE0-GD3BJ and CAM-B3LYP-GD3BJ calculated maximum absorption wavelengths (λ, nm) and oscillator strengths (*f*) in THF for L^F^ and related [RuL^F^_3-n_(bpy)_n_]^2+^ complexes. Table S12: Computed dipole moments (Debye) for the ground S0 and vertical S1 states.

## Sample Availability

Samples of the compounds are not available from the authors.

## Abbreviations

OPA: One-photon absorption, TPA: two-photon absorption, TPEF: two-photon excited fluorescence, CT: charge transfer, ILCT: intra-ligand charge transfer, MLCT: metal-to-ligand charge transfer, LMCT: ligand-to-metal charge transfer, LLCT: ligand-to-ligand charge transfer, MO: molecular orbital, HOMO: Highest Occupied Molecular Orbital, LUMO: Lowest Unoccupied Molecular Orbital, DFT: density functional theory, TD-DFT: time-dependent density functional theory.
