# Supplementary material for: Two-Photon Absorption Cooperative Effects within Multi-Dipolar Ruthenium Complexes: The Decisive Influence of Charge Transfers
Source: Molecules. 2022 Feb 23;27(5):1493. doi: 10.3390/molecules27051493 (PMC8912064; doi:10.3390/molecules27051493)
Supplement: Supplementary file 1 [file molecules-27-01493-s001.zip › molecules-1587692-supplementary.pdf]

Two-Photon Absorption Cooperative Effects within Multi-dipolar Ruthenium Complexes: The Decisive Influence of Charge Transfers

Nicolas Durand, Anissa Amar, Rana Mhanna, Huriye Akdas-Kiliç, Olivier Soppera, Jean-Pierre Malval, Abdou Boucekkine*, Jean-Luc Fillaut*.

Supporting information

Page 2-3: Synthesis and main characterizations.
Page 4:
Figure S1. UV-vis absorption spectra of L^C and $[Ru(L^C)_{3-n}(bpy)_n]^{2+}$ (n = 0-2) complexes;
Figure S2. UV-vis absorption spectra of L^F and $[Ru(L^F)_{3-n}(bpy)_n]^{2+}$ (n = 0-2) complexes;
Pages 5-6:
Figure S3. Emission spectra of L^T and $[Ru(L^T)_{3-n}(bpy)_n]^{2+}$ (n = 0-2) complexes;
Figure S4. Emission spectra of L^C and $[Ru(L^C)_{3-n}(bpy)_n]^{2+}$ (n = 0-2) complexes;
Figure S5. Emission spectra of L^F and $[Ru(L^F)_{3-n}(bpy)_n]^{2+}$ (n = 0-2) complexes;
Page 6 : Scheme S1. Simplified Jablonski diagram for two-photon excitations in $[Ru(bpy)_3]^{2+}$-like complexes
Page 7:
Figure S6. TPA spectra of L^C and $[Ru(L^C)_{3-n}(bpy)_n]^{2+}$ (n = 0-2) complexes;
Figure S7. TPA spectra of L^F and $[Ru(L^F)_{3-n}(bpy)_n]^{2+}$ (n = 0-2) complexes;
Page 8: . Scheme S2 : Main charge transfers upon excitation within $Ru(L^T)_3$ as a model of complexes containing extended π-system ligands
Page 9 : Examples of calculation of the effective number of electrons involved in two-photon transitions
Page 10:
Figure S8. Energy diagram of the frontier MOs of the ligands.
Pages 11-12:
Figure S9 : main MOs of $[Ru(L^T)_3]^{2+}$
Figure S10 : main MOs of $[Ru(L^C)_3]^{2+}$
Figure S11 : main MOs of $[Ru(L^F)_3]^{2+}$
Figure S12: Density differences plots (Dρ(r)= ρS1(r)- ρS0(r)) of of L and related [RuL3-n(bpy)n]2+ complexes (n = 0-2), between S1–S0 states (red=increase, blue=decrease of electron density; isovalue 0.03 au).

Page 13:
Table S1. Main structural characteristics of L and related $[RuL_{3-n}(bpy)_n]^{2+}$ complexes
Pages 14-15:
Table S2 : Energies of the frontier MOs of bipyridines L = L^T, L^C, L^F
Table S3 : Energies and percentage contributions of the frontier MOs of $[RuL^T{}_{3-n}(bpy)_n]^{2+}$ complexes
Table S4 : Energies and percentage contributions of the frontier MOs of $[RuL^C{}_{3-n}(bpy)_n]^{2+}$ complexes
Table S5 : Energies and percentage contributions of the frontier MOs of $[RuL^F{}_{3-n}(bpy)_n]^{2+}$ complexes
Pages 16-19:
Table S6. PBE0-GD3BJ and CAM-B3LYP-GD3BJ simulated UV-visible spectra of L^T and related $[RuL^T{}_{3-n}(bpy)_n]^{2+}$ complexes
Table S7. PBE0-GD3BJ/Lanl2DZP and CAM-B3LYP-GD3BJ/Lanl2DZP Simulated UV-visible spectra of L^C and related $[RuL^C{}_{3-n}(bpy)_n]^{2+}$ complexes
Table S8. PBE0-GD3BJ/Lanl2DZP and CAM-B3LYP-GD3BJ/Lanl2DZP Simulated UV-visible spectra of L^F and related $[RuL^F{}_{3-n}(bpy)_n]^{2+}$ complexes
Pages 20-23:
Table S9. PBE0-GD3BJ and CAM-B3LYP-GD3BJ calculated maximum absorption wavelengths (λ, nm) and oscillator strengths (*f*) in THF for L^T and related $[RuL^T{}_{3-n}(bpy)_n]^{2+}$ complexes.
Table S10. PBE0-GD3BJ and CAM-B3LYP-GD3BJ calculated maximum absorption wavelengths (λ, nm) and oscillator strengths (*f*) in THF for L^C and related $[RuL^C{}_{3-n}(bpy)_n]^{2+}$ complexes

Table S11. PBE0-GD3BJ and CAM-B3LYP-GD3BJ calculated maximum absorption wavelengths (λ, nm) and oscillator strengths (*f*) in THF for L^F and related $[RuL^F_{3-n}(bpy)_n]^{2+}$ complexes).

Table S12. Computed dipole moments (Debye) for the ground S0 and vertical S1 states

Synthesis and main characterizations.

Most manipulations were performed using Schlenk techniques under an Ar atmosphere. All solvents were dried and purified by standard procedures. The bipyridyl ligands L^T, L^C, L^F were synthesized following previously reported procedures.[46,58] NMR spectra were recorded on Bruker DPX-200, AV 300 or AV 500 MHz spectrometers. ^{1}H and ^{13}C chemical shifts are given versus $SiMe_4$ and were determined by reference to residual ^{1}H and ^{13}C solvent signals. UV–vis absorption spectra were recorded using a UVIKON 9413 or Biotek Instruments XS spectrophotometer using quartz cuvettes of 1 cm path length. Steady-state luminescence spectra were measured using a Jobin Yvon FluoroMax-2 or Tau-3 spectrofluorimeter. The two-photon absorption (TPA) measurements were performed with femtosecond mode-locked laser pulse using a Ti: Sapphire laser (Coherent, Chameleon Ultra II: pulse duration: ~140 fs; repetition rate: 80 MHz; wavelength range: 680-1040 nm). Electrospray ionization mass spectrometric (ESI–MS) measurements have been made on a Bruker Daltonics Amazon SL ion trap and Agilent Q–TOF MS mass spectrometer. Elemental analyses were performed at the Centre de Mesures Physiques de l'Ouest (CRMPO) in Rennes.

$[RuL^T(bpy)_2]^{2+}$, $2(PF_6^-)$: Yield: 73 mg, 36 %. ^{1}H NMR (400 MHz, CD_2Cl_2) δ 8.54 (s, 2H), 8.46 (d, *J* = 6 Hz, 4H), 8.10 (t, *J* = 7.1 Hz, 4H), 7.86 (d, *J* = 5.6 Hz, 2H), 7.75 (d, *J* = 5.6 Hz, 2H), 7.59 – 7.48 (m, 14H), 7.34 (t, *J* = 5.6 Hz, 8H), 7.17 – 7.12 (m, 14H), 7.27 (d, *J* = 5.6 Hz, 4H). ^{13}C NMR (101 MHz, CD_2Cl_2) δ 156.66, 156.49, 151.43, 149.28, 147.54, 146.91, 137.79, 129.48, 128.86, 128.63, 125.26, 123.94, 123.75, 121.92, 121.15. MALDI-TOF-MS: calc. for ($[C_{70}H_{54}N_8F_6PRu]^+$) *m/z* = 1253.3157, found *m/z* = 1257.729 Anal. Calcd. For $[C_{70}H_{54}N_8F_{12}P_2Ru]$: C, 60.13; H, 3.89; N, 8.01. Found: C, 59.77; H, 3.86; N, 7.90.

$[RuL^C(bpy)_2]^{2+}$, $2(PF_6^-)$: Yield: 144.7 mg, 30 %. ^{1}H NMR (400 MHz, acetone-d6) δ 9.11 (s, 2H), 8.88 (d, *J* = 8 Hz, 4H), 8.53 (s, 2H), 8.26 – 8.23 (m, 8H), 8.13 (d, *J* = 8 Hz, 2H), 8.05 (d, *J* = 16 Hz, 2H), 7.95 (d, *J* = 8 Hz, 2H), 7.90 (dd, *J* = 4 Hz, 8 Hz, 2H), 7.75 – 7.60 (m, 12H), 7.51 (d, *J* = 16 Hz, 2H), 7.30 (t, *J* = 8 Hz, 2H), 4.53 (t, *J* = 8 Hz, 4H), 1.95 (m, 4H), 1.45 – 1.23 (m, 20H), 0.88 (t, *J* = 8 Hz, 6H). ^{13}C NMR (101 MHz, acetone-d6) δ 157.42, 157.34, 157.30, 151.81, 151.81, 151.61, 151.19, 147.70, 141.43, 141.11, 137.97, 137.92, 127.91, 127.84, 126.92, 126.31, 125.41, 124.39, 123.87, 123.31, 122.65, 120.99, 120.56, 120.31, 120.21, 119.52, 109.71, 109.59, 109.43, 42.78, 31.59, 29.7, 29.5, 29.1, 26.90, 22.34, 13.41. MALDI-TOF-MS: calc. for ($[C_{74}H_{74}N_8F_6PRu]^+$) *m/z* = 1321.4722, found *m/z* = 1322.404. Anal. Calcd. For $[C_{74}H_{74}N_8F_{12}P_2Ru, 0.5CH_2Cl_2]$: C, 59.28; H, 5.01; N, 7.43. Found: C, 59.32; H, 5.52; N, 7.07.

$[RuL^F(bpy)_2]^{2+}$, $2(PF_6^-)$: Yield: 20 mg, 12 %. ^{1}H NMR (400 MHz, CD_2Cl_2) δ 8.54 (d, *J* = 2.0 Hz, 2H), 8.46 (d, *J* = 8.2 Hz, 6H), 8.08 (t, *J* = 7.9 Hz, 6H), 7.85 (dd, *J* = 5.8, 1.3 Hz, 2H), 7.75 (d, *J* = 5.6 Hz, 2H), 7.69 (d, *J* = 4.3 Hz, 2H), 7.66 – 7.59 (m, 6H), 7.54 – 7.47 (m, 10H), 7.27 (t, *J* = 7.7 Hz, 10H), 7.10 (d, 6H), 7.03 (m, *J* = 6.7, 6.1 Hz, 6H), 2.02 – 1.81 (m, 8H), 1.25 – 1.03 (m, 40H), 0.83 (t, *J* = 6.9 Hz, 12H), 0.68 (s, 8H). ^{13}C NMR (101 MHz, CD_2Cl_2) δ 156.8, 156.8, 156.7, 152.8, 151.5, 151.2, 151.2, 150.7, 148.0, 147.8, 147.8, 147.4, 143.2, 137.9, 137.9, 137.8, 135.1, 133.4, 129.1, 128.0, 127.9, 127.3, 124.1, 124.1, 124.0, 123.1, 122.7, 122.1, 121.7, 120.7, 120.7, 119.4, 118.8, 55.1, 40.1, 31.7, 29.9, 29.3, 29.2, 23.9, 22.6, 13.8. MALDI-TOF-MS: calc. for ($[C_{116}H_{126}N_8F_6PRu]^+$) *m/z* = 1877.8791, found *m/z* = 1879.2596. Anal. Calcd. For $[C_{116}H_{126}N_8F_{12}P_2Ru],CH_2Cl_2$: C, 66.64; H, 6.12; N, 5.32. Found: C, 66.62; H, 5.97; N, 5.21.

General synthetic procedure for the Ru(L)$_2$(bpy)(PF$_6$)$_2$ complexes: Ru(bpy)Cl$_2$(DMSO)$_2$ (35 mg, 0.072 mmol) and the bipyridine L (2 eq. , 0.144 mmol) were heated at 90°C for two days in degassed dimethylformamide (3 mL) under argon. The products were treated and purified as indicated above to give the expected products as red powders.

[Ru(L^T)$_2$(bpy)]$^{2+}$, 2(PF$_6^-$): Yield: 45 mg, 68 %. ^{1}H NMR (300 MHz, CD$_2$Cl$_2$) δ 8.42 (s, 4H), 8.36 (d, *J* = 8.8 Hz, 2H), 7.90 (t, *J* = 6.8 Hz, 2H), 7.80 (d, *J* = 6.8 Hz, 2H), 7.57 (d, *J* = 6.8 Hz, 2H),7.50 – 7.41 (m, 20H), 7.35 – 7.26 (m, 20H), 7.28 – 7.23 (m, 24H), 6.95 (d, *J* = 6.8 Hz, 8H). ^{13}C NMR (125 MHz, CD$_2$Cl$_2$) δ 156.78, 149.00, 146.70, 135.62, 129.39, 129.32, 129.32, 128.64, 128.32, 128.32, 125.30, 124.97, 124.97, 123.85, 123.85, 123.53, 121.38, 121.38, 121.28, 119.98. MALDI-TOF-MS: calc. for ([C$_{110}$H$_{84}$N$_{10}$F$_6$PRu]$^+$) *m/z* = 1791.5166, found *m/z* = 1793.365. Anal. Calcd. For [C$_{110}$H$_{84}$N$_{10}$F$_{12}$P$_2$Ru], 1.5 CH$_2$Cl$_2$: C, 64.75; H, 4.26; N, 6.81. Found: C, 64.36; H, 4.67; N, 6.83.

[Ru(L^C)$_2$(bpy)]$^{2+}$, 2(PF$_6^-$): Yield: 96 mg, 74 %. ^{1}H NMR (400 MHz, acetone-d6) δ 9.13 (s, 4H), 8.90 (d, *J* = 8 Hz, 2H), 8.55 (d, *J* = 8 Hz, 4H), 8.29-8.20 (m, 8H), 8.11 (d, *J* = 8 Hz, 2H), 8.08 (d, *J* = 8 Hz, 2H), 8.04 (d, *J* = 8 Hz, 2H), 7.97 (d, *J* = 4 Hz, 2H), 7.92 (t, *J* = 8 Hz, 4H), 7.79-7.74 (m, 4H), 7.72-7.62 (m, 10H), 7.56-7.48 (m, 8H), 7.30-7.25 (m, 4H), 4.53 (m, 8H), 1.94 (m, 8H), 1.42-1.24 (m, 40H), 0.87 (t, *J* = 8 Hz, 12H). ^{13}C NMR (101 MHz, acetone-d6) δ 151.22, 141.44, 141.12, 126.94, 126.32, 125.40, 123.32, 122.63, 121.06, 120.51, 120.29, 119.53, 109.72, 42.78, 31.59, 31.57, 26.91, 22.35, 22.33, 13.42, 13.40. MALDI-TOF-MS: calc. for ([C$_{118}$H$_{124}$N$_{10}$F$_6$PRu]$^+$) *m/z* = 1927.8696, found *m/z* = 1929.8841. Anal. Calcd. For [C$_{118}$H$_{124}$N$_{10}$F$_{12}$P$_2$Ru]: C, 68.36; H, 6.03; N, 6.76. Found: C, 67.44; H, 5.86; N, 6.94.

[Ru(L^F)$_2$(bpy)]$^{2+}$, 2(PF$_6^-$): Yield: 33 mg, 29 %. ^{1}H NMR (400 MHz, CD$_2$Cl$_2$) δ 8.62 (s, 4H), 8.51 (d, *J* = 9 Hz, 2H), 8.14 (t, *J* = 9 Hz, 2H), 7.93 (d, *J* = 6.8 Hz, 2H), 7.77 (d, *J* = 6.8 Hz, 2H), 7.67 – 7.52 (m, 30H),7.38 – 7.27 (m, 20H), 7.17 (m, 20H), 7.10 – 7.02 (m, 10H), 2.02 – 1.81 (m, 16H), 1.25 - 1.03 (m, 80H), 0.83 (t, *J* = 6.9 Hz, 24H), 0.68 (s, 16H). ^{13}C NMR (101 MHz, CD$_2$Cl$_2$) δ 157.09, 152.86, 151.51, 150.73, 147.98, 147.88, 143.20, 137.59, 135.24, 133.54, 129.20, 127.30, 124.06, 123.20, 122.77, 120.78, 119.46, 118.93, 40.18, 31.80, 29.97, 29.32, 29.22, 23.92, 22.61, 18.35. MALDI-TOF-MS: calc. for ([C$_{202}$H$_{228}$N$_{10}$F$_6$PRu]$^+$) *m/z* = 3042.6906, found *m/z* = 3044.0561. Anal. Calcd. For [C$_{202}$H$_{228}$N$_{10}$F$_{12}$P$_2$Ru], 1.5 CH$_2$Cl$_2$: C, 73.73; H, 7.03; N, 4.23. Found: C, 73.18; H, 7.22; N, 4.12.

General synthetic procedure for the Ru(L)$_3$,(PF$_6$)$_2$ complexes: RuCl$_2$(DMSO)$_4$ (20.5 mg, 0.042 mmol) and the bipyridine L (0.131 mmol) were heated at 90°C for one day and 130°C for two days in 3 mL degassed dimethylformamide, under argon. The products were treated and purified as indicated above to give the expected products as red powders.

[Ru(L^T)$_3$]$^{2+}$, 2(PF$_6^-$): Yield: 55 mg, 54 %. ^{1}H NMR (400 MHz, CD$_2$Cl$_2$) δ 8.55 (s, 6H), 7.66 (d, *J* = 5.8 Hz, 6H), 7.56 (d, *J* = 16.0 Hz, 6H), 7.53 (d, *J* = 7.6 Hz, 12H), 7.46 (d, *J* = 5.8 Hz, 6H), 7.33 (t, J = 7.1 Hz, 24H), 7.15 (m, 24H), 7.14 (m, 12H), 7.13 (d, *J* = 16.0 Hz, 6H), 7.04 (d, *J* = 7.6 Hz, 12H). ^{13}C NMR (101 MHz, CD$_2$Cl$_2$) δ 156.99, 150.29, 149.49, 147.20, 146.92, 136.43, 129.45, 128.70, 128.46, 125.38, 124.00, 123.46, 121.73, 121.69, 120.95. MALDI-TOF-MS: calc. for ([C$_{150}$H$_{114}$N$_{12}$F$_6$PRu]$^+$) *m/z* = 2329.7975, found *m/z* = 2329.2915.Anal. Calcd. For [C$_{150}$H$_{114}$N$_{12}$F$_{12}$P$_2$Ru], CH$_2$Cl$_2$: C, 70.83; H, 4.57; N, 6.56. Found: C, 70.23; H, 4.47; N, 6.74.

[Ru(L^C)$_3$]$^{2+}$, 2(PF$_6^-$): Yield: 68 mg, 60 %. ^{1}H NMR (400 MHz, acetone-d6) δ 9.20 (s, 6H), 8.51 (s, 6H), 8.20 (d, *J* = 6 Hz, 5H), 8.17 (d, *J* = 9 Hz, 5H), 8.10 (d, *J* = 15 Hz, 6H), 7.91 (d, *J* = 9 Hz, 5H), 7.84 (d, *J* = 6 Hz, 7H), 7.73 (d, *J* = 6 Hz, 6H), 7.69 (d, *J* = 9 Hz, 6H), 7.55 (d, *J* = 6 Hz, 7H), 7.53 (d, *J* = 15 Hz, 6H), 7.31 (t, *J* = 6 Hz, 7H), 4.53 (t, *J* = 6 Hz, 12H), 1.93 (q, *J* = 7.1 Hz, 12H), 1.48 – 1.22 (m, 60H), 0.90 (d, *J* = 7.0 Hz, 18H).^{13}C NMR (101 MHz, acetone-d6) δ 147.41, 141.42, 141.10, 127.13, 126.97, 125.37, 123.31, 122.63, 120.28, 119.52, 109.73, 109.61, 42.79, 31.68, 31.58, 26.90, 22.40, 22.34, 13.41, 13.25. MALDI-TOF-MS: calc. for ([C$_{162}$H$_{174}$N$_{12}$F$_6$PRu]$^+$) *m/z* = 2534.2670, found *m/z* = 2538.8442. Anal. Calcd. For [C$_{162}$H$_{174}$N$_{12}$F$_{12}$P$_2$Ru].2C$_7$H$_{16}$.3.5CH$_2$Cl$_2$: C, 67.88; H, 6.76; N, 5.30. Found: C, 68.77; H, 6.68; N, 5.28.

[Ru(L^F)$_3$]$^{2+}$, 2(PF$_6^-$): Yield: 41 mg, 38 %. ^{1}H NMR (400 MHz, CD_2Cl_2) δ 8.65 (s, 6H), 7.81 – 7.60 (m, 48H), 7.40 – 7.28 (m, 36H), 7.17 – 7.03 (m, 36H), 2.02 – 1.80 (m, 24H), 1.25 - 1.03 (m, 120H), 0.83 (t, *J* = 8 Hz, 36H), 0.68 (s, 24H). ^{13}C NMR (101 MHz, CD_2Cl_2) δ 157.08, 152.89, 151.56, 147.99, 147.88, 147.16, 143.21, 137.60, 135.23, 133.52, 129.20, 127.30, 124.07, 123.93, 123.20, 122.78, 121.77, 120.79, 119.46, 118.93, 40.17, 31.80, 29.97, 29.68, 29.31, 29.21, 23.91, 22.61, 13.85. MALDI-TOF-MS: calc. for ([$C_{288}H_{330}N_{12}F_6PRu$]$^+$) *m/z* = 4206.4983, found *m/z* = 4206.2824. Anal. Calcd. For [$C_{288}H_{330}N_{12}F_{12}P_2Ru$], 1.5 CH_2Cl_2: C, 77.63; H, 7.50; N, 3.76. Found: C, 77.20; H, 7.80; N, 3.46.

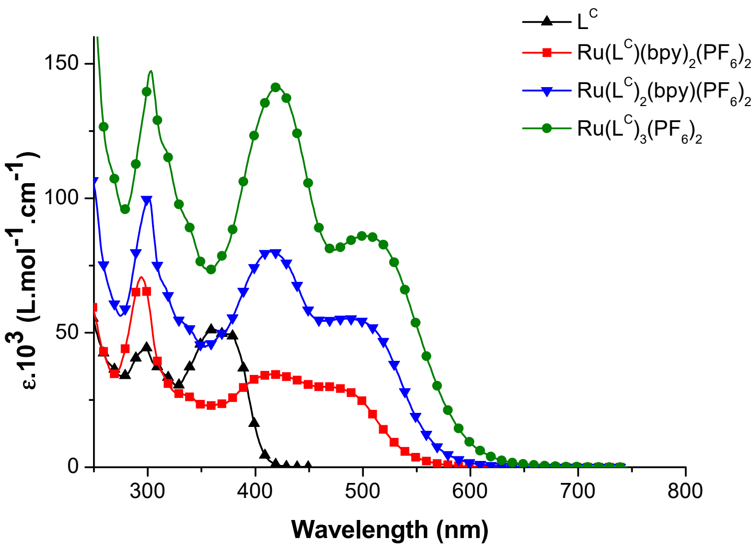

Figure S1. UV-vis absorption spectra of L^C and [Ru(L^C)$_{3-n}$(bpy)$_n$]$^{2+}$ (n = 0-2) complexes; THF ; c = 1.0 x 10^{-5} mol.L^{-1}, 25 °C.

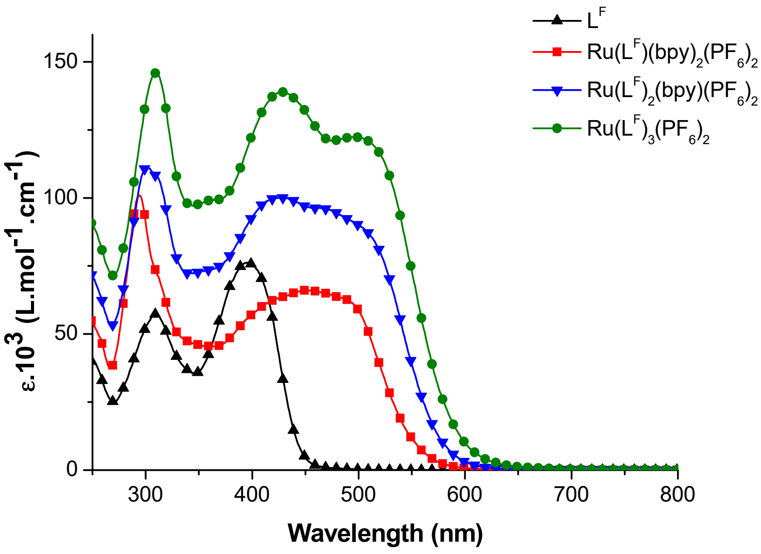

Figure S2. UV-vis absorption spectra of L^F and [Ru(L^F)$_{3-n}$(bpy)$_n$]$^{2+}$ (n = 0-2) complexes; THF ; c = 1.0 x 10^{-5} mol.L^{-1}, 25 °C.

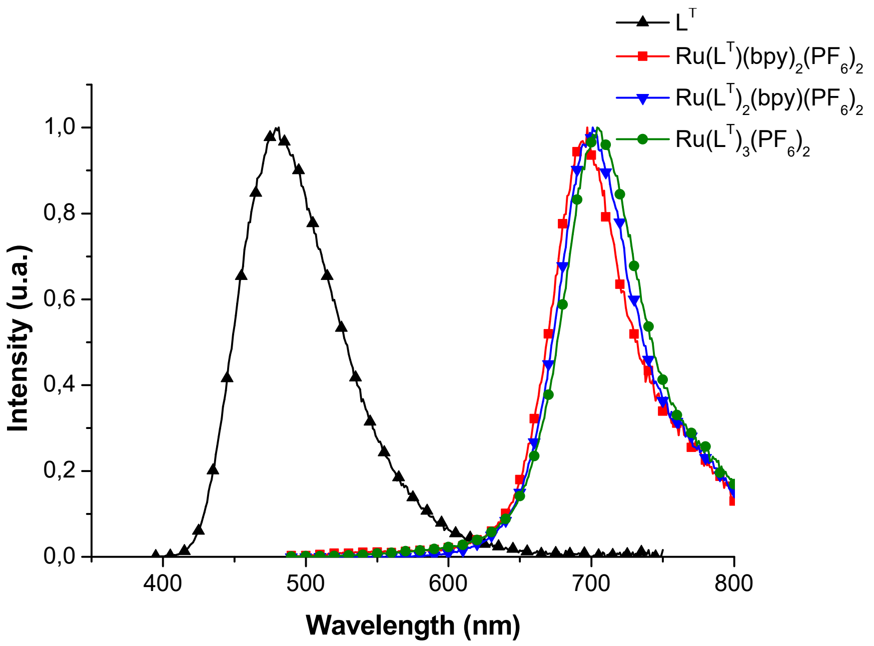

Figure S3. Emission spectra of L^T and $[Ru(L^T)_{3-n}(bpy)_n]^{2+}$ (n = 0-2) complexes; degassed THF ; c = 1.0 x 10^{-5} mol.L^{-1}, 25 °C.

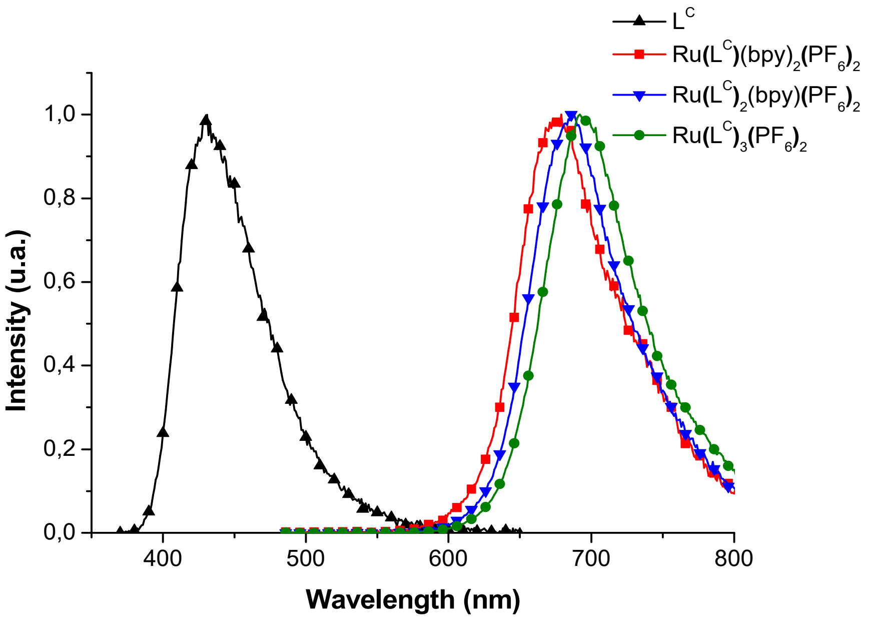

Figure S4. Emission spectra of L^C and $[Ru(L^C)_{3-n}(bpy)_n]^{2+}$ (n = 0-2) complexes; degassed THF ; c = 1.0 x 10^{-5} mol.L^{-1}, 25 °C.

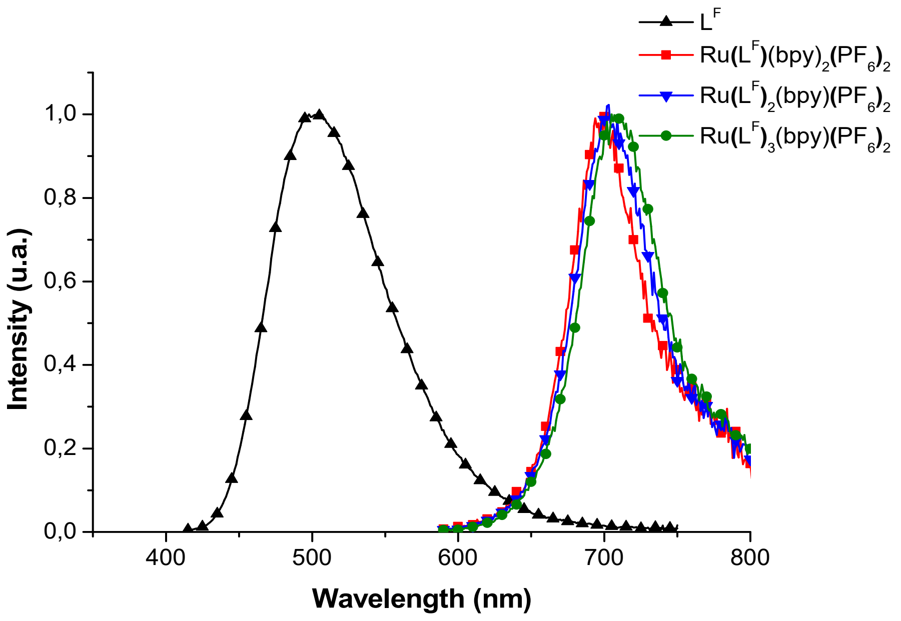

Figure S5. Emission spectra of L^F and $[Ru(L^F)_{3-n}(bpy)_n]^{2+}$ (n = 0-2) complexes; degassed THF ; c = 1.0 x 10^{-5} mol.L^{-1}, 25 °C.

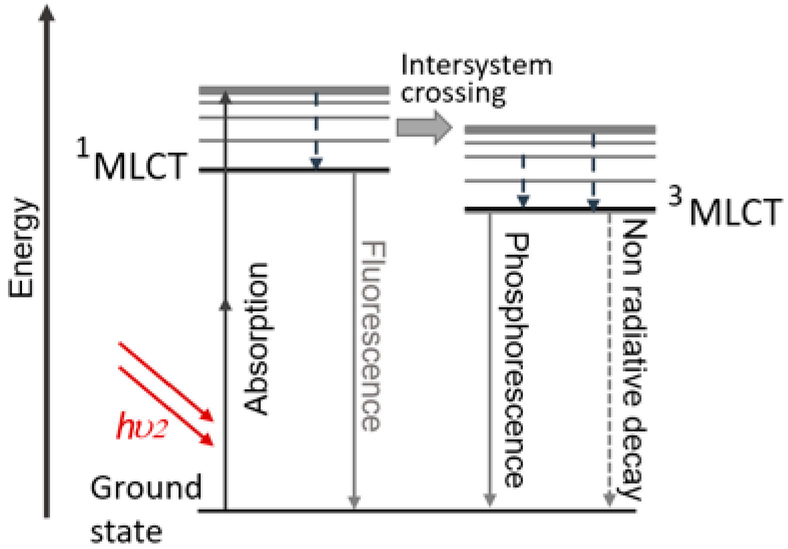

Scheme S1. Simplified Jablonski diagram for two-photon excitations in $[Ru(bpy)_3]^{2+}$-like complexes. Two-photon excitation occurs between the ground state and the vibrational levels of the first electronic excited state. After excitation process, the fluorophore relaxes to the lowest energy level of the first excited electronic states ^{1}MLCT via vibrational processes, followed by intersystem crossing to the lowest triplet state of the complexes from where emission occurs. The subsequent emission processes for both one- and two-photon excitations are thus the same.

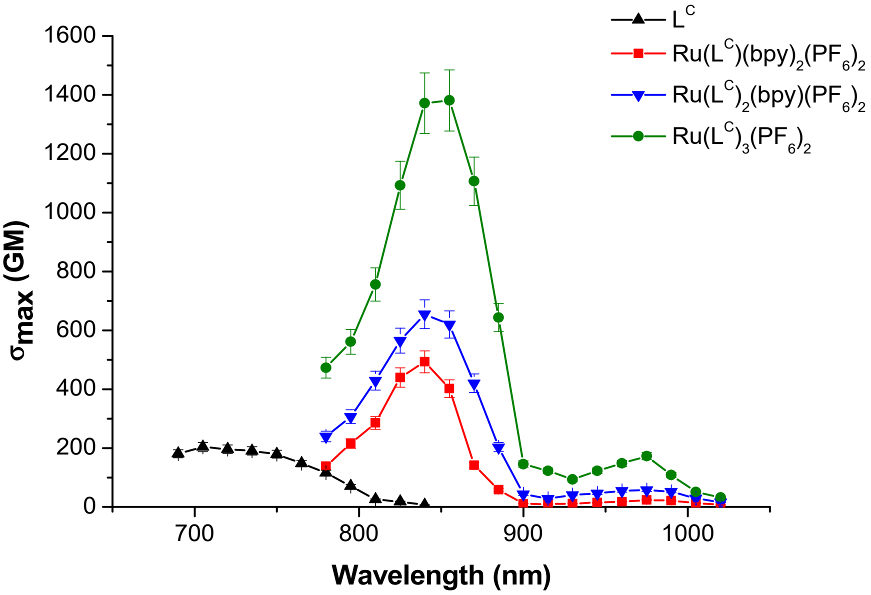

Figure S6. TPA spectra of L^C and $[Ru(L^C)_{3-n}(bpy)_n]^{2+}$ (n = 0-2) complexes; THF; c = 1.0 x 10^{-5} mol.L^{-1}, 25 °C.

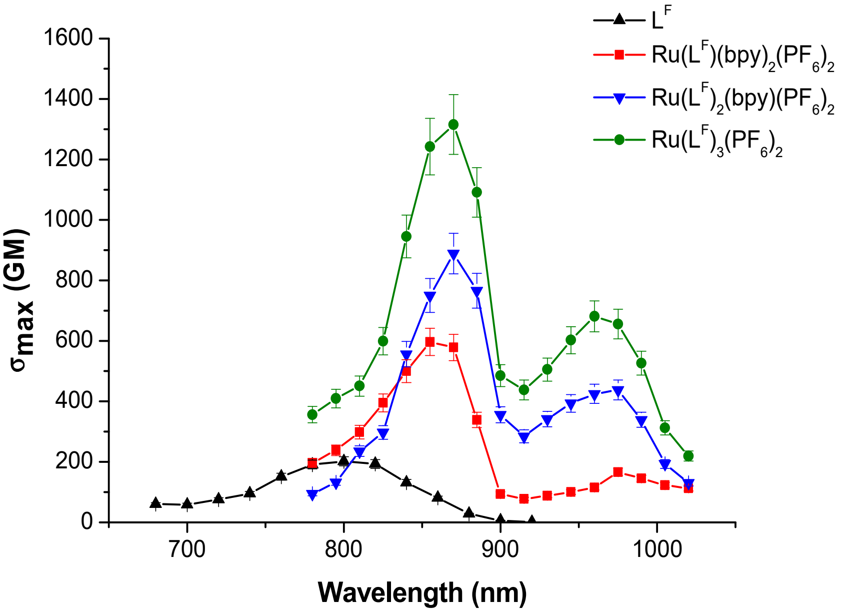

Figure S7. TPA spectra of L^F and $[Ru(L^F)_{3-n}(bpy)_n]^{2+}$ (n = 0-2) complexes; THF; c = 1.0 x 10^{-5} mol.L^{-1}, 25 °C.

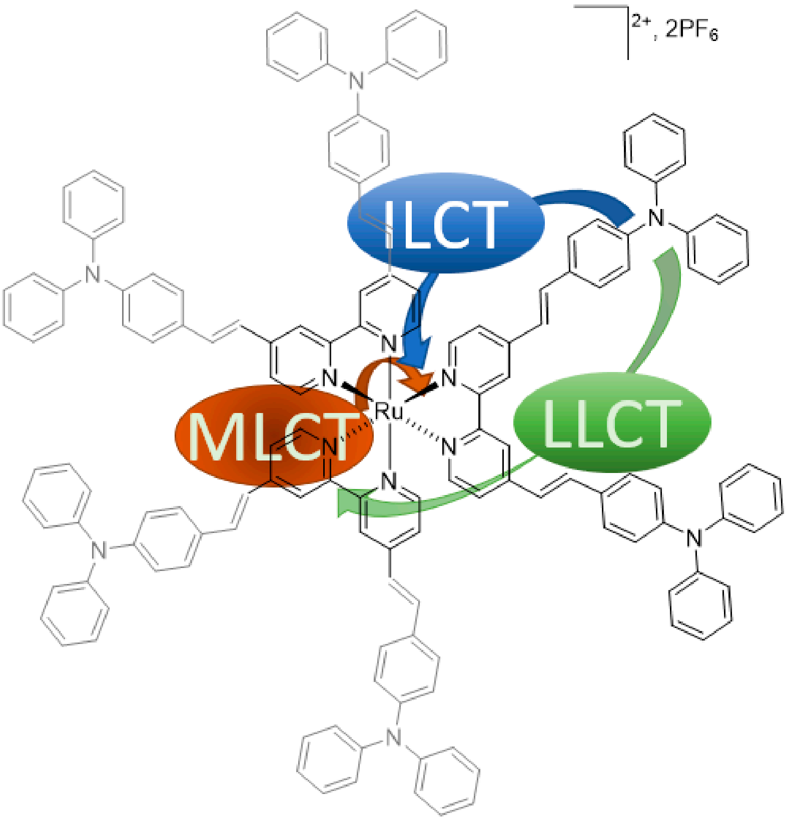

Scheme S2. Main charge transfers upon excitation within Ru(L^T)$_3$ as a model of complexes containing extended π-system ligands

Examples of calculation of the effective number of electrons involved in two-photon transitions.

Ligand L^T :

$$\delta'_{max} = \frac{\delta_{max}}{N_{eff}^2} \qquad N_{eff} = \sqrt{\sum_i N_i^2}$$

N= number of π -electron in the conjugated part of the molecule

$$N_{eff} = \sqrt{A * B^2 + C^2 * D} = 23.1$$

A= 2, number of donor -acceptor parts

B= 14, number of π -electrons in a donor- acceptor part

C= 6, number of π -electrons in a phenyl moiety

D= 4, number of phenyl units per molecule

$[Ru(L^T)(bpy)_2]^{2+}$:

N= number of π-electrons in the conjugated part of the molecule

$$N_{eff} = \sqrt{A * B^2 + C^2 * D + E^2 * F + G^2} = 28.7$$

A= 2, number of donor acceptor parts

B= 14, number of π -electrons in a donor- acceptor part

C= 6, number of π -electrons in a phenyl moiety

D= 4, number of phenyl units in the molecule

E= 6, number of π -electrons in a pyridine moiety

F= 4, number of pyridine units in the molecule

G= 12, number of electrons in $[Ru(L^T)(bpy)_2]^{2+}$ bonds

Kuzyk, M. G. Fundamental Limits on Two-Photon Absorption Cross Sections. *J. Chem. Phys.* **2003**, *119* (16), 8327–8334. https://doi.org/10.1063/1.1611474.

Moreno, J. P.; Kuzyk, M. G. Fundamental Limits of the Dispersion of the Two-Photon Absorption Cross Section. *J Chem Phys* **2005**, *123* (19), 194101. https://doi.org/10.1063/1.2104407.

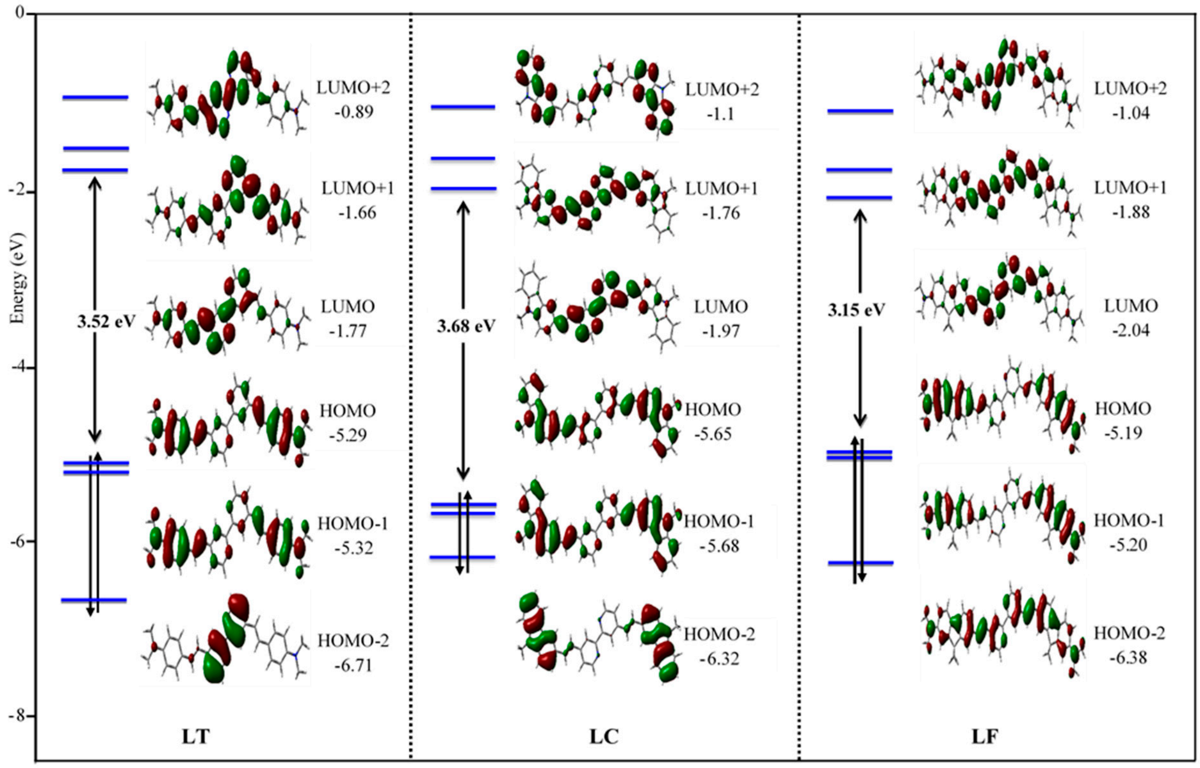

Figure S8. Energy diagram of the frontier MOs of the ligands.

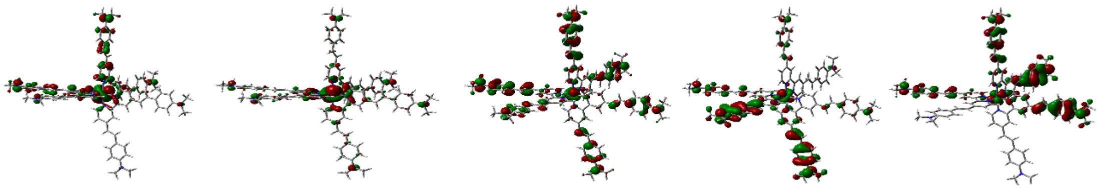

HOMO-7 HOMO-6 HOMO-2 HOMO-1 HOMO

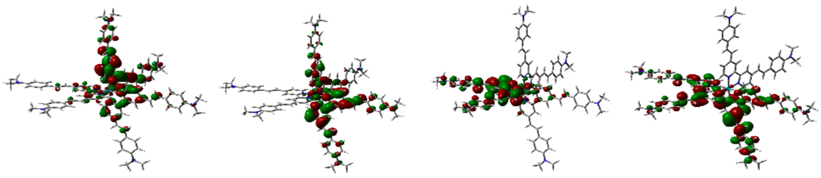

LUMO LUMO+1 LUMO+2 LUMO+3

Figure S9 : main MOs of $[Ru(L^T)_3]^{2+}$

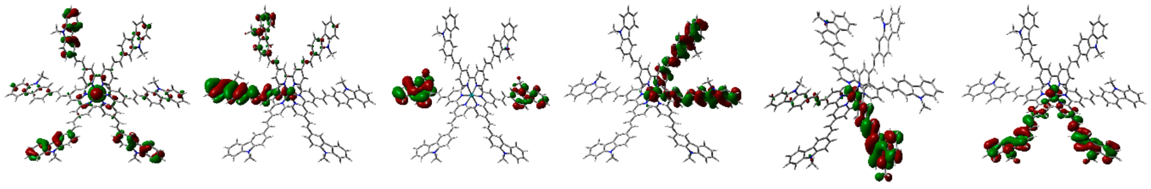

HOMO-12 HOMO-7 HOMO-4 HOMO-3 HOMO-1 HOMO

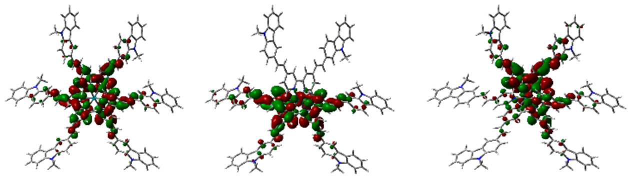

LUMO LUMO+1 LUMO+2

Figure S10 : main MOs of $[Ru(L^C)_3]^{2+}$

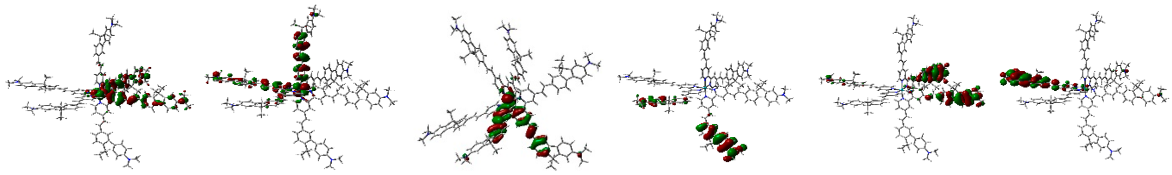

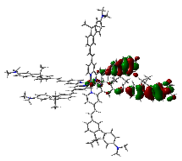

HOMO-9 HOMO-8 HOMO-6 HOMO-3 HOMO-2 HOMO-1
HOMO

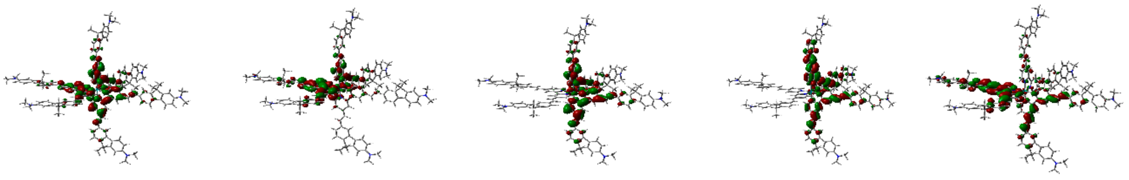

LUMO LUMO+1 LUMO+2 LUMO+3 LUMO+4

Figure S11 : main MOs of $[Ru(L^F)_3]^{2+}$

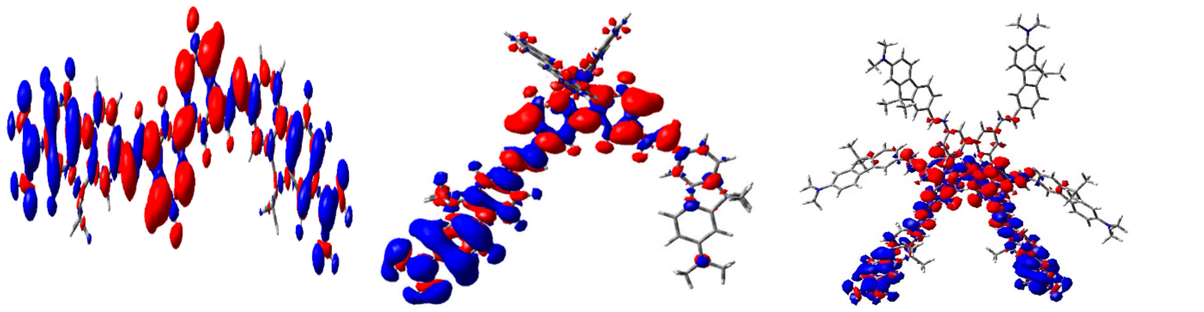

q^{CT} = 0.853 e d^{CT} = 1.31 Å | q^{CT} = 1.07 e d^{CT} = 7.406 Å | q^{CT} = 1.065 e d^{CT} = 5.641 Å

L^F | **$[Ru(L^F) (bpy)_2]^{2+}$** | **$[Ru(L^F)_3]^{2+}$**

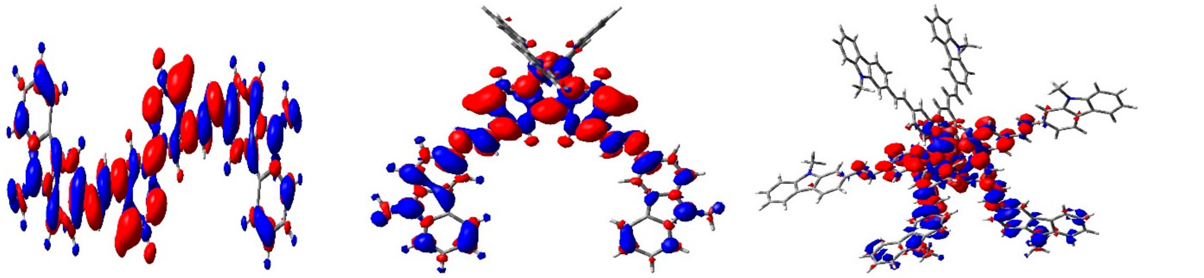

L^C | **$[Ru(L^C) (bpy)_2]^{2+}$** | **$[Ru(L^C)_3]^{2+}$**

q^{CT} = 0.68e d^{CT} = 0.006 Å | q^{CT} = 0.78 e d^{CT} = 3.19 Å | q^{CT} = 0.83e d^{CT} = 2.60 Å

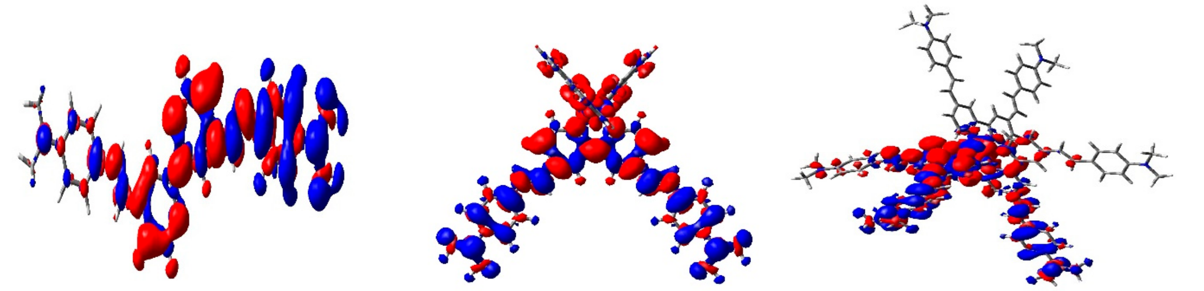

q^{CT} = 0.66 e d^{CT} = 3.60 Å | q^{CT} = 0.80 e d^{CT} = 3.91 Å | q^{CT} = 0.84 e d^{CT} = 2.67 Å

L^T | **$[Ru(L^T) (bpy)_2]^{2+}$** | **$[Ru(L^T)_3]^{2+}$**

Figure S12: Density differences plots ($D\rho(r) = \rho^{S1}(r) - \rho^{S0}(r)$) of of L and related $[RuL_{3-n}(bpy)_n]^{2+}$ complexes (n = 0-2), between S_1–S_0 states (red=increase, blue=decrease of electron density; isovalue 0.03 au).

Table S1. Main structural characteristics of $[RuL_{3-n}(bpy)_n]^{2+}$ complexes (n = 0-2).

		$[Ru(L^C)(bpy)_2]^{2+}$	$[Ru(L^C)_3]^{2+}$	$[Ru(L^F)(bpy)_2]^{2+}$	$[Ru(L^F)_3]^{2+}$	$[Ru(L^T)(bpy)_2]^{2+}$	$[Ru(L^T)_3]^{2+}$
Bond lengths (Å)	Ru-N1	2.060	2.058	2.060	2.058	2.060	2.057
	Ru-N2	2.060	2.057	2.060	2.058	2.060	2.057
	Ru-N3	2.058	2.057	2.058	2.056	2.058	2.056
	Ru-N4	2.060	2.057	2.059	2.057	2.059	2.058
	Ru-N5	2.059	2.057	2.060	2.057	2.059	2.057
	Ru-N6	2.058	2.056	2.058	2.056	2.058	2.057
Bond angles (°)	N1-Ru-N2	78.58	78.58	78.57	78.59	78.53	78.62
	N2-Ru-N3	88.37	88.07	88.18	88.30	88.16	88.76
	N3-Ru-N4	78.90	78.62	78.88	78.61	78.88	78.68
	N4-Ru-N5	87.96	88.18	88.05	88.37	88.02	88.03
	N5-Ru-N6	78.88	78.63	78.87	78.63	78.88	78.59
	N6-Ru-N1	88.26	88.09	87.72	88.39	88.32	88.37

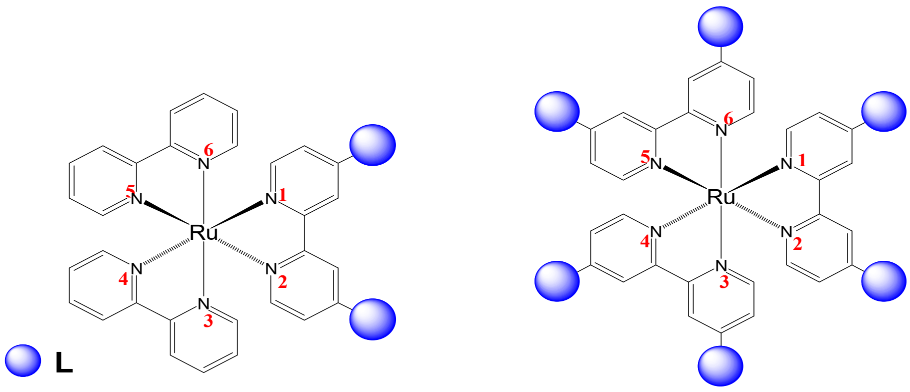

$[Ru(L)_3]^{2+}$ $[Ru(L)(bipy)_2]^{2+}$

Table S2: Energies of the frontier MOs of bipyridines L = L^T, L^C, L^F

	HOMO-4	HOMO-3	HOMO-2	HOMO-1	HOMO	LUMO	LUMO+1	LUMO+2	LUMO+3
L^T	-6.99	-6.92	-6.71	-5.32	-5.29	-1.77	-1.66	-0.89	-0.21
L^C	-6.77	-6.32	-6.32	-5.68	-5.65	-1.97	-1.76	1.1	-1.06
L^F	-6.81	-6.40	-6.38	-5.20	-5.19	-2.04	-1.88	-1.04	-0.52

Table S3 : Energies and percentage contributions of the frontier MOs of $[RuL^T_{3-n}(bpy)_n]^{2+}$ complexes (n = 0 ; 2).

$[Ru(L^T)(bpy)_2]^{2+}$										
	HOMO-7	HOMO-6	HOMO-4	HOMO-2	HOMO-1	HOMO	LUMO	LUMO+1	LUMO+2	LUMO+3
Energy (eV)	-7.80	-7.80	-6.98	-6.81	-5.83	-5.82	-3.10	-2.98	-2.95	-2.39
Ru (%)	1	0	66	66	9	18	7	20	11	0
L^T (%)	81	76	3	22	69	64	8	1	73	78
$(bipy)_2$ (%)	0	0	27	8	6	3	77	74	6	1
$[Ru(L^T)_3]^{2+}$										
Energy (eV)	-6.62	-6.48	-5.78	-5.68	-5.67	-5.67	-2.92	-2.78	-2.72	-2.36
Ru (%)	54	65	0	10	11	11	1	6	5	1
L^T_1 (%)	20	13	52	28	43	11	12	4	93	48
L^T_2 (%)	28	11	15	34	8	46	68	24	3	1
L^T_3 (%)	5	11	27	23	35	26	21	67	7	63

Table S4 : Energies and percentage contributions of the frontier MOs of $[RuL^{C}_{3-n}(bpy)_n]^{2+}$ complexes (n = 0 - 2).

$[Ru(L^C)(bpy)_2]^{2+}$														
	HOMO-12	HOMO-7	HOMO-6	HOMO-5	HOMO-4	HOMO-3	HOMO-2	HOMO-1	HOMO	LUMO	LUMO+1	LUMO+2	LUMO+3	LUMO+9
Energy (eV)	-8.43	-7.48	-7.04	-7.02	-6.90	-6.72	-6.71	-6.14	-6.13	-3.19	-3.04	-2.99	-2.53	-1.50
Ru (%)	1	8	69	48	64	7	7	4	5	2	4	6	1	0
L^C (%)	93	88	10	41	24	79	92	93	92	78	18	0	98	93
$(bipy)_2$ (%)	2	2	20	8	10	1	1	1	1	20	78	94	0	0
$[Ru(L^C)_3]^{2+}$														
Energy (eV)	-6.79	-6.32	-6.29	6.29	-6.29	-6.23	-6.14	-6.07	-6.04	-3.20	-3.07	-3.07	-2.54	-1.47
Ru(%)	36	8	0	0	0	21	1	10	12	1	6	6	1	0
L^C_1 (%)	21	65	0	17	78	2	46	6	44	32	55	8	1	32
L^C_2 (%)	20	16	75	1	1	28	3	80	1	34	2	56	48	37
L^C_3 (%)	19	1	2	79	17	41	48	2	42	32	37	27	46	27

Table S5 : Energies and percentage contributions of the frontier MOs of $[RuL^{F}_{3-n}(bpy)_n]^{2+}$ complexes (n = 0 ; 2).

	$[Ru(L^F)(bpy)_2]^{2+}$												
	HOMO-8	HOMO-7	HOMO-6	HOMO-5	HOMO-4	HOMO-3	HOMO-2	HOMO-1	HOMO	LUMO	LUMO+1	LUMO+2	LUMO+3
Energy (eV)	-7.39	-7.27	-7.28	-7.15	-6.99	-6.72	-6.71	-5.53	-5.52	-3.20	-3.04	-2.98	-2.59
Ru (%)	3	49	36	43	66	32	43	1	1	2	4	6	1
LF (%)	94	22	51	43	16	60	49	92	92	83	13	0	98
$(bipy)_2$ (%)	1	28	7	10	18	5	6	0	0	15	83	94	0
	$[Ru(L^F)_3]^{2+}$												
Energy (eV)	-6.48	-6.46	-6.38	-5.56	-5.55	-5.55	-5.51	-5.49	-5.48	-3.16	-3.04	-3.03	-2.51
Ru (%)	43	42	44	0	0	0	0	0	0	0	6	6	0
L^F_1 (%)	2	18	25	3	77	14	5	87	0	33	61	2	1
L^F_2 (%)	26	2	4	88	1	0	47	3	44	32	25	34	55
L^F_3 (%)	26	21	26	1	13	79	42	2	48	33	7	55	38

Table S6. PBE0-GD3BJ and CAM-B3LYP-GD3BJ simulated UV-visible spectra of L^T and related $[RuL^T_{3-n}(bpy)_n]^{2+}$ complexes (n = 0 ; 2).

	PBE0-GD3BJ	CAM-B3LYP-GD3BJ
L^T	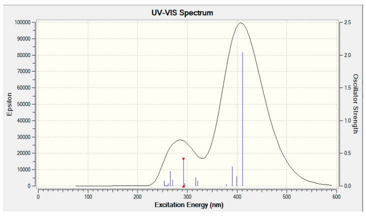	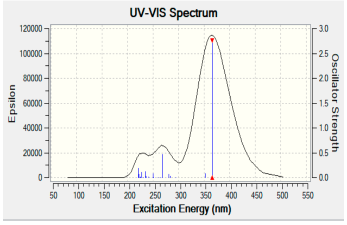
$[Ru(L^T)(bpy)_2]^{2+}$	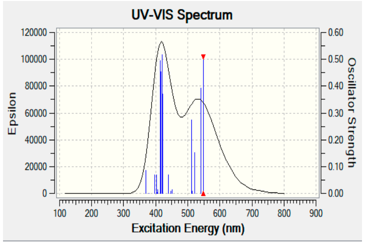	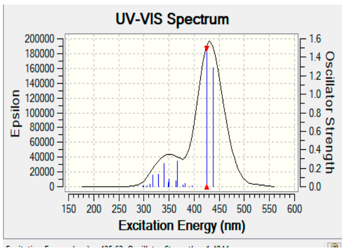
$[Ru(L^T)_3]^{2+}$	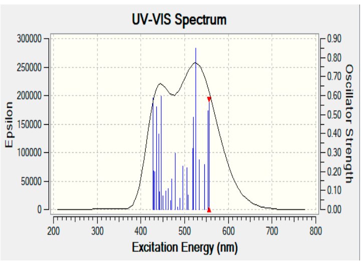	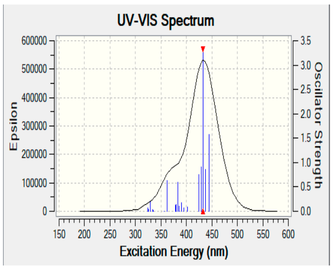

Table S7. PBE0-GD3BJ/Lanl2DZP and CAM-B3LYP-GD3BJ/Lanl2DZP Simulated UV-visible spectra of L^C and related $[RuL^C_{3-n}(bpy)_n]^{2+}$ complexes (n = 0 ; 2).

<table>
<tr><th></th><th>PBE0-GD3BJ</th><th>CAM-B3LYP-GD3BJ</th></tr>
<tr><td>L^C</td><td>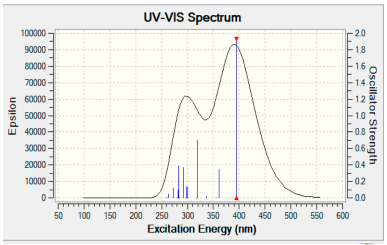
</td><td>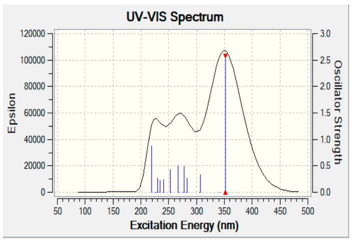
</td></tr>
<tr><td>$[Ru(L^C)(bpy)_2]^{2+}$</td><td>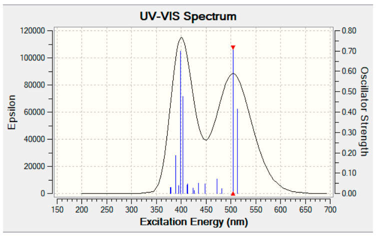
</td><td>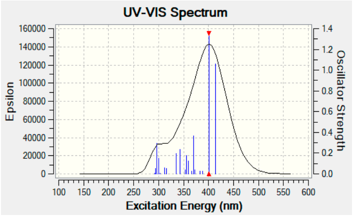
</td></tr>
<tr><td>$[Ru(L^C)_3]^{2+}$</td><td>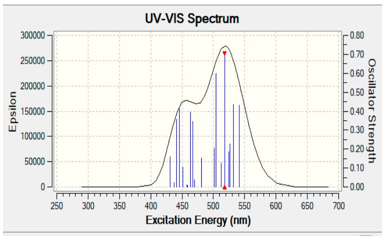
</td><td>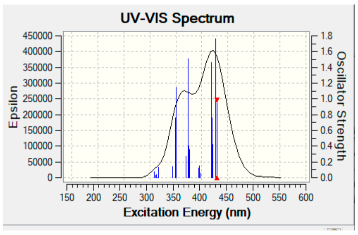
</td></tr>
</table>

Table S8. PBE0-GD3BJ/Lanl2DZP and CAM-B3LYP-GD3BJ/Lanl2DZP Simulated UV-visible spectra of L^F and related $[RuL^F_{3-n}(bpy)_n]^{2+}$ complexes (n = 0 ; 2).

	PBE0-GD3BJ	CAM-B3LYP-GD3BJ
L^F	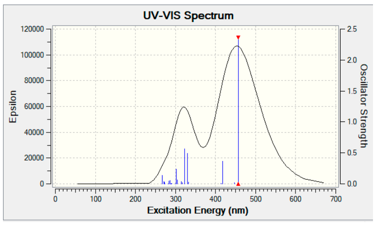	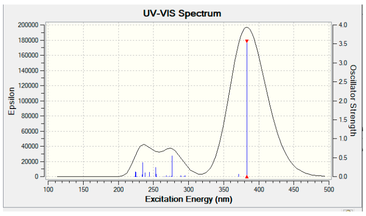
$[Ru(L^F)(bpy)_2]^{2+}$	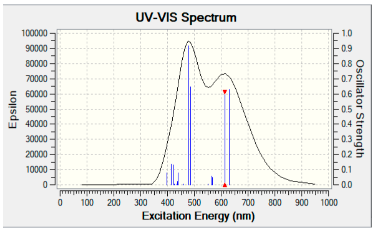	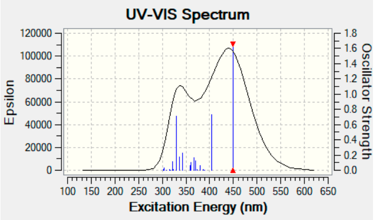
$[Ru(L^F)_3]^{2+}$	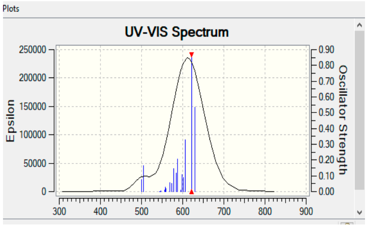	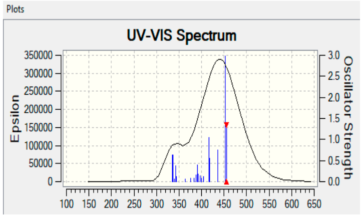

Table S9. PBE0-GD3BJ and CAM-B3LYP-GD3BJ calculated maximum absorption wavelengths (λ, nm) and oscillator strengths (*f*) in THF for L^T and related $[RuL^T_{3-n}(bpy)_n]^{2+}$ complexes (n = 0 ; 2).

		λ_{max}(nm)	λ_{calc} (*f*)	Main transitions (weight)
1GL^T	PBE0-GD3BJ	411	411 (2.04)	H-1->L (56%); H->L (21%); H->L+1(20%)
		285	293 (0.41)	H-2->L (73%); H-4->L (9%)
	CAM-B3LYP-GD3BJ	365	365 (2.77)	H-1->L (41%); H->L+1(39%)
		268	268 (0.47)	H-2->L (62%); H-1->L (12%)
$[Ru(L^T)(bpy)_2]^{2+}$	PBE0-GD3BJ	530	548 (0.51)	H->L (88%)
			541 (0.40)	H-1->L (94%)
			511 (0.30)	H-1->L+1(89%)
		415	420 (0.52)	H-1->L+3(37%); H-4->L (34%)
	CAM-B3LYP-GD3BJ	430	437 (1.30)	H->L (61%); H-1->L+3(19%)
			426 (1.50)	H-1->L (64%); H->L+3(14%)
		350	367(0.30)	H-2->L (48%); H-4->L+1(29%)
$[Ru(L^T)_3]^{2+}$	PBE0-GD3BJ	525	557 (0.60)	H->L(66%); H-2->L(18%)
			553 (0.52)	H-2->L(59%); H->L(21%)
			525(0.85)	H-1->L+1 (66%)
		447	447(3.30)	H-1-> L+3 (60%); H-2-> L+3 (17%)
	CAM-B3LYP-GD3BJ	434	444(1.60)	H->L(47%); H-7->L(10%)
			433 (3.32)	H->L+1(13%); H-1->L+2(11%)
		370	383 (0.60)	H-6->L(22%); H-3->L+1(10%)

λ_{calc} are the TD-DFT computed wavelengths ; λ_{max} are the maximum of the simulated bands

Table S10. PBE0-GD3BJ and CAM-B3LYP-GD3BJ calculated maximum absorption wavelengths (λ, nm) and oscillator strengths (*f*) in THF for L^C and related $[RuL^C_{3-n}(bpy)_n]^{2+}$ complexes (n = 0 ; 2).

		λ_{max}(nm)	λ_{calc} (*f*)	Main transitions (weight)
L^C	PBE0-GD3BJ	390	395 (1.93)	H-1->L (74%); H->L+1(23%)
		297	319 (0.70)	H-3->L (+40%); H-1->L+2(21%); H->L+3(18%)
	CAM-B3LYP-GD3BJ	350	352 (2.58)	H-1->L (43%); H->L+1(41%)
		270	278 (0.49)	H-4 -> L (19%); H-3->L+2 (17%); H-2->L+1(15%)
$[Ru(L^C)(bpy)_2]^{2+}$	PBE0-GD3BJ	500	513 (0.42)	H->L (50%); H-1->L (36%)
			504 (0.72)	H-1->L (52%); H->L(39%)
		400	403 (0.48)	H->L+3 (45%); H-1->L+3(29%)
			399(0.70)	H-1->L+3(42%); H->L+3(24%); H-6->L+2(11%)
	CAM-B3LYP-GD3BJ	400	414 (1.05)	H-1->L (48%); H->L+3(15%); H-4->L(15%)
			401 (1.35)	H-1->L(48%); H-5->L(14%)
		300	296(0.30)	H->L+9(41%); H-3->L(14%) ; H-3->L+9(13%)
$[Ru(L^C)_3]^{2+}$	PBE0-GD3BJ		541 (0.43)	H->L (72%)
		518	532 (0.44)	H-1 -> L (57%)
			518(0.70)	H-3->L (67%); H-1->L+2(10%)
			504 (0.60)	H-1->L+1 (30%); H-3->L+2(23%); H->L+1 (16%)
		455	467(0.34)	H-7->L (40%); H-2->L+1(26%)
			463 (0.40)	H-2->L+1 (48%); H-7->L (19%)
	CAM-B3LYP-GD3BJ	420	429 (0.99)	H->L (28%); H->L+2(14%)
			427(1.76)	H-1->L (24%); H-1->L+2(14%)
		360	373 (1.50)	H-12->L (18%); H-4->L+2(5%)

Table S11. PBE0-GD3BJ and CAM-B3LYP-GD3BJ calculated maximum absorption wavelengths (λ, nm) and oscillator strengths (*f*) in THF for L^F and related $[RuL^F{}_{3-n}(bpy)_n]^{2+}$ complexes (n = 0 ; 2).

		λ_{max}(nm)	λ_{calc} (*f*)	Main transitions (weight)
L^F	PBE0-GD3BJ	455	458 (2.36)	H-1->L (71%); H->L+1(25%)
		323	330 (0.49)	H-1->L+2 (81%)
			323 (0.56)	H-3->L (52%); H-2->L+1 (13%); H-2->L (11%)
	CAM-B3LYP-GD3BJ	383	383 (3.57)	H-1->L (39%); H->L+1(39%)
		276	276 (0.54)	H-4->L (49%); H-1>L (25%)
$[Ru(L^F)(bpy)_2]^{2+}$	PBE0-GD3BJ	615	631 (0.63)	H->L (94%)
			615 (0.61)	H-1->L (96%)
		479	479(0.91)	H->L+3(91%)
	CAM-B3LYP-GD3BJ	430	450 (1.66)	H->L (56%); H->L+3(18%); H-3->L (14%)
			404 (0.73)	H-1->L (42%); H-2->L (27%); H-1->L+3(11%)
		335	330 (0.71)	H-2->L+3(21%); H-1->L (14%); H-5->L (11%); H-6->L (11%)
$[Ru(L^F)_3]^{2+}$	PBE0-GD3BJ	605	630 (0.54)	H->L (84%)
			622(0.87)	H-1->L (79%)
		504	504 (0.17)	H-6->L (80%)
	CAM-B3LYP-GD3BJ	435	455 (1.34)	H->L (26%); H-2-> L+2 (14%); H-8->L (12%); H->L+1(11%)
			453 (2.97)	H-1->L (19%); H-1->L+1(18%)
		340	335 (0.64)	H-3->L (15%); H->L(8%); H-9->L+1(+6%); H-6->L+4(+6%); H->L+3(5%)

Table S12 : Computed dipole moments (Debye) for the ground S0 and vertical S1 states

	$[Ru(L^T)_3]^{2+}$	$[Ru(L^C)_3]^{2+}$	$[Ru(L^F)_3]^{2+}$
μ_{S0}	0.30	0.03	1.81
μ_{S1}	8.60	4.30	13.19
$\Delta\mu$	8.30	4.27	11.38
